# How do people with multimorbidity prioritise healthcare when faced with tighter financial constraints? A national survey with a choice experiment component

**DOI:** 10.1186/s12875-025-02738-9

**Published:** 2025-02-27

**Authors:** James Larkin, Louise Foley, Shane Timmons, Tony Hickey, Barbara Clyne, Patricia Harrington, Susan M. Smith

**Affiliations:** 1 Department of General Practice, RCSI University of Medicine and Health Sciences, Dublin, Ireland; 2 School of Allied Health and Health Research Institute, University of Limerick, Limerick, Ireland; 3 Behavioural Research Unit, Economic and Social Research Institute, Dublin, Ireland; 4 Multimorbidity Patient and Public Involvement Group, National University of Ireland Galway, Galway, Ireland; 5 Health Information and Quality Authority, Dublin, Ireland; 6 Department of Public Health & Epidemiology, School of Population Health, RCSI University of Medicine and Health Sciences, Dublin, Ireland; 7 Centre for Health Policy & Management, School of Medicine, Trinity College Dublin, Dublin, Ireland

**Keywords:** Chronic disease, Financial burden, Health care costs, Multimorbidity, Non-communicable disease

## Abstract

**Background:**

People with multimorbidity (i.e., two or more chronic conditions) experience increased out-of-pocket healthcare costs and are vulnerable to cost-related non-adherence to recommended treatment. The aim of this study was to understand how people with multimorbidity prioritise different healthcare services when faced with tighter budget constraints and how they experience cost-related non-adherence.

**Methods:**

A national cross-sectional online survey incorporating a choice experiment was conducted. Participants were adults aged 40 years or over with at least one chronic condition, recruited in Ireland (December 2021 to March 2022). The survey included questions about real-life experiences of cost-related non-adherence and financial burden. The choice experiment element involved participants identifying how they would prioritise their real-world healthcare utilisation if their monthly personal healthcare budget was reduced by 25%.

**Results:**

Among the 962 participants, 64.9% (*n* = 624) had multimorbidity. Over one third (34.5%, *n* = 332) of participants reported cost-related non-adherence in the previous 12 months, which included not attending a healthcare appointment and/or not accessing medication. Similar findings on prioritisation were observed on the choice task. When presented with the hypothetical tighter budget constraint, participants reduced expenditure on ‘other healthcare (hospital visits, specialist doctors, etc.)’ by the greatest percentage (50.2%) and medicines by the lowest percentage (24.8%). Participants with multimorbidity tended to have a condition they prioritised over others. On average, they reduced expenditure for their top-priority condition by 71% less than would be expected if all conditions were valued equally, while they reduced expenditure for their least prioritised condition by 60% more than would be expected. Independence, symptom control and staying alive were rated as the most important influencing factors when making prioritisation decisions (median score = 5 out of 5).

**Conclusion:**

When faced with tighter financial constraints, people with multimorbidity tended to have a condition they prioritised over others. Participants were also more likely to prioritise medicines over other aspects of healthcare. Researchers, policymakers and clinicians should take greater consideration of the different ways people respond to tighter financial constraints. This could involve reducing the payment barriers to accessing care or clinicians discussing healthcare costs and coverage with patients as part of cost-of-care conversations.

**Supplementary Information:**

The online version contains supplementary material available at 10.1186/s12875-025-02738-9.

## Introduction

People with two or more chronic conditions (i.e., multimorbidity) often experience high out-of-pocket healthcare costs [1–5]. These costs sometimes mean people must forego other expenses (e.g., necessities like groceries or social spending), but they can also force people to sacrifice some aspects of their healthcare [6, 7]. Foregoing aspects of healthcare due to tighter budget constraints is referred to as ‘cost-related non-adherence’ to healthcare recommendations [6, 7]. Cost-related non-adherence is risky for health and wellbeing [8], but also generates significant challenges for healthcare systems [9]. Given rising prevalence rates of multimorbidity in recent decades [2–5] and the association between multimorbitity and lower socio-economic status [10], understanding how people with multimorbidity make trade-offs in their healthcare utilisation when faced with tighter budget constraints is crucial for informing policy responses.

Previous research shows that high out-of-pocket healthcare costs lead people with multimorbidity to employ a range of coping strategies [11, 12], including cost-related non-adherence to recommended healthcare [6, 7]. The literature investigating cost-related non-adherence has thus far focused primarily on how and why people prioritise between *medications* when faced with tighter budget constraints [13]. Some show that people prioritise short-term benefits (e.g. symptom relief) over long-term benefits (e.g. reduced risk of mortality) [14], whereas others show the reverse [15]. Others show that non-adherence choices may be condition dependent, with medications associated with either mental health disorders or respiratory conditions more likely to be de-prioritised [6].

However, people faced with tighter budget constraints may also choose to sacrifice areas of healthcare other than medication (e.g., not attending GP or hospital appointments). We could locate no study that has examined how people make trade-offs between different areas of their healthcare when faced with tighter budget constraints, despite important implications for policy and care.

This study addressed this gap in two ways. First, we surveyed people with chronic health conditions about their health expenditure and used this data to create a novel, individualised choice experiment. The choice experiment imposed a hypothetical tighter budget constraint on their individual expenses and participants were asked to re-allocate this constrained budget across their typical healthcare utilisation. Hypothetical choice experiments allow for decision-making scenarios to be simulated under highly controlled conditions, permitting choices that might otherwise have serious real-world consequences to be recorded under more ethical settings. The goal here was to shed light on how people with multimorbidity might prioritise healthcare (both medications and other forms) if forced to prioritise, because of tighter budget constraints. Another advantage of the choice experiment is that it facilitates an understanding of the behaviours of people who have not experienced a specific scenario but may experience that scenario in the future.

Hypothetical experiments, however, have limitations. As such, our second approach to investigating broader forms of cost-related non-adherence was to implement a survey, asking participants about their lived experience of financial burden associated with managing their healthcare and what influences their decisions if faced with tighter budgetary constraints. Thus, we provide evidence on (1) how people with multimorbidity might prioritise healthcare when faced with tighter budget constraints that necessitate doing so and (2) how people with multimorbidity have managed with real-life tighter budget constraints in the past.

We collected our data in Ireland and, in any investigation of healthcare costs, the individual policy context matters, specifically the healthcare entitlements system. Healthcare entitlements are the rights or benefits that facilitate individuals accessing the healthcare system, generally by wholly or partly reducing financial charges for accessing healthcare services. We describe the policy context for Ireland and provide an overview of the healthcare entitlements system in Box 1.

**Box 1:** Healthcare entitlements in Ireland

Ireland has a mixed entitlements system: there are publicly funded programmes to increase access to healthcare aimed at those with low incomes and/or high healthcare need. There are three main healthcare entitlement categories: ‘basic eligibility’, General Practitioner (GP) Visit Card (partial eligibility) and Medical Card (full eligibility) [16]. These three categories are mutually exclusive.
- ‘**Basic eligibility**’ entitles people to access the hospital system though with a range of co-payments. At the time of data collection there were upper-limits on charges for inpatient care (€80 per day and €800 annually), emergency department attendance (€100 per visit) and prescription medicines (€114 per month at end of 2021, €100 per month at start of 2022) [17, 18]. Patients must pay out-of-pocket for GP visits, with no upper limit. The average cost of a GP visit in Ireland is €54 [19].
- For the **GP Visit Card**, basic eligibility applies but there is also coverage for GP consultation fees [20]. Those under 70 with household incomes below a threshold are entitled to a GP visit card, with all adults aged over 70 years eligible [16, 21]. As an example of this threshold, a single person living alone aged under 66 years would be entitled to the GP visit card if their weekly income was below €304 (less tax) [22].
- The **Medical Card** is a means-tested scheme which provides free primary, community and hospital care, and heavily subsidised prescription medicines [23]. The income threshold for adults for the Medical Card at the time of data collection was approximately 40% lower than the GP visit card threshold [16].
In 2020, 35% of people in Ireland had a Medical Card, 11% had a GP Visit Card and 54% had basic eligibility [24]. Approximately 46% of the population have private health insurance [25], which facilitates access to private care but generally does not cover GP care. Others pay the full cost out-of-pocket for private care.

## Aim

This study aimed to understand how people with multimorbidity prioritise their healthcare when experiencing tighter budget constraints. To achieve this aim, there were two objectives: first, to understand how people with multimorbidity choose between different healthcare services and chronic conditions when presented with hypothetical tighter budget constraints; second, to understand their real-life experiences of healthcare prioritisation.

## Methods and data

The study design was a national cross-sectional online survey conducted in Ireland. The survey included two primary elements that all participants completed: (1) A choice experiment in which people with chronic conditions, including those with a single condition and those with multimorbidity, were asked to prioritise their previous month’s healthcare utilisation within the context of hypothetical tighter budget constraints; and (2) Survey questions about real-life experiences of cost-related non-adherence and financial burden. The study is reported according to the STROBE guidelines [26].

### Participants

The inclusion criteria were: (a) aged 40 years or over, (b) self-reported as having one or more chronic conditions, (c) access to a functional broadband connection and (d) access to a computer, laptop, tablet or smart-phone. The age group of 40 years and over was chosen to increase the likelihood of identifying people who meet the inclusion criteria, as chronic conditions and multimorbidity are less common in those aged under 40 years [27]. People with one condition were included to allow for comparison with those with multimorbidity, in line with previous studies of multimorbidity and out-of-pocket healthcare expenditure [2]. People with zero chronic conditions were not included as people with zero conditions utilise less healthcare [3] and therefore a larger proportion of them would be unable to complete the choice experiment. People were excluded if they: were pregnant or had been pregnant in the previous three months; or had an insufficient level of English language proficiency required to take part. People who were pregnant were excluded as they were likely to have had different patterns of recent healthcare utilisation associated with their pregnancy.

### Sampling

Data collection was carried out by Behaviour and Attitudes, an independent survey company [28]. The company was tasked with sampling a group representative of the public aged 40 years and over with one or more conditions in terms of age, region, sex, socioeconomic status, and area (urban/rural) using stratified random sampling. The company primarily recruited from their pre-established national online research panel of 27,951 people aged 16 years or over. Given there were no patient safety concerns, and no appropriate data on which to base a sample size calculation, we sought to maximise the sample size in order to provide results as representative of the populations as possible. The survey was conducted remotely, online by participants on their own electronic device. The online questionnaire was developed using Askia software. Panel members were sent an initial email (Appendix B, eBox 3) briefly describing the survey and providing a link to an online portal to participate.

Although the online panel presents an efficient way to collect large samples, the necessity to be a regular internet user generates selection bias. Internet penetration in Ireland is very high, with 92% of households having internet access [29]. However, certain groups report not using the internet in a given three month period, particularly those aged 75 years and over (56%) and the socioeconomically most disadvantaged quintile (16%) [30]. To increase the response rate among these groups, we supplemented the panel with door-to-door recruitment. Researchers from the survey company conducted random door-to-door sampling in randomly chosen locations in Ireland. When going door-to-door (details in Appendix B, eBox 5), recruiters first requested demographic information (Appendix C). Then, if the prospective participant met the inclusion criteria and were in an under-represented group (Appendix D, eTable 1), they were provided with study information and details of how it could be completed online (Appendix B, eBox 6). Nonetheless, the necessity to complete the survey on an electronic device with internet access represents a significant barrier to completing the survey for older adults.

Participants were offered €10 to complete the survey. Participants who did not complete the survey within 17 days of initial contact were sent a reminder email (Appendix B, eBox 4). Three more reminders were sent, each seven days after the previous email. Data was collected between December 2021 and March 2022. The survey did not contact new prospective participants between 24th December-16th January because they may have had altered patterns of healthcare usage during the December holiday period.

If participants exited the survey before completion, this was deemed a non-response and answers were not included in analysis. The survey company noted participants’ IP address and device type to prevent people completing the survey twice. This data was temporarily stored by Behaviour and Attitudes until data collection was completed and was only accessible to named individuals at Behaviour and Attitudes.

### Survey design and structure

The survey design was initially informed by a review of Piette and colleagues’ conceptual framework for understanding individuals’ responses to medication costs [13]. The survey design was also informed by reviewing literature in the areas of multimorbidity [31, 32] out-of-pocket healthcare expenditure [33, 34], survey methodology [35] and behavioural economics [36] (further details below). It was then revised based on feedback from a panel of people with multimorbidity and by a pilot (both described below). Overall, there were between 59 and 107 questions, depending on the number of health conditions a participant reported having. Participants were initially asked for health, demographic and entitlement information, following that they completed the choice experiment and were then asked questions about their real-life experiences of financial burden (further details below). Average survey completion-time was 19 min. All questions required a response. A back button was provided to allow participants to change responses. Question types included a mix of closed and open-ended responses. The closed-ended questions were either Likert scale questions or multiple-choice questions. The full questionnaire can be seen in Appendix A.

### Health, demographic and entitlement information

Participants were presented with a list of 31 chronic conditions which was based on the list used in a national survey of older adults [37]. If a participant detailed a condition under ‘other (please specify)’, this person was included only if they had at least one other chronic condition listed in the survey, as it was not feasible to prepare choice experiment questions for conditions reported under the ‘other’ category. Participants were then asked demographic and healthcare entitlement questions.

### Choice experiment: overview and rationale

The next component of the survey was the choice experiment. A choice experiment is a survey approach whereby participants are presented with several options and the choices they make are used to identify their preferences [38]. This choice experiment involved participants first providing their previous month’s healthcare utilisation and associated out-of-pocket expenditure across four different healthcare areas (GP, prescription medicines, primary care, and ‘other healthcare (e.g. hospital visits, specialist doctors, etc.)’). Participants were then presented with a hypothetical tighter budget constraint based on their individual spend and were asked to decide which areas of their healthcare they would reduce their spending in. The task made it salient that, when faced with a tighter budget constraint, retaining the same level of spending in one area would require reductions in spending in another.

The experiment was not a standard ‘discrete’ choice experiment. Discrete choice experiments involve presenting participants with multiple scenarios that vary systematically by levels of specified attributes. Their preferred scenarios over multiple rounds of choices are used to estimate the weight they assign to those attributes and the levels within them [39]. The distinction here is that trade-offs between different attributes are implicit, whereas our design overcomes this limitation and more closely mimics a situation in which people might be required to forego some healthcare utilisation due to tighter budget constraints.

### Choice experiment: healthcare utilisation

Participants were asked to approximate their healthcare utilisation and the associated out-of-pocket costs for the previous month, broken down into four categories: GP, prescription medicines, ‘primary care (e.g. physio, occupational therapists, psychologists, etc)’, and ‘other healthcare (e.g. hospital visits, specialist doctors, etc.)’. These four categories were chosen as they represent the majority of out-of-pocket healthcare costs experienced by households in Ireland [34]. Participants were also asked to break down their expenditure by condition. ‘Multiple/other conditions’ was included as a condition to allow for healthcare utilisation that applies to multiple conditions or other health issues. Participants were asked to include travel expenses in all out-of-pocket healthcare expenditure estimates.

### Choice experiment: healthcare expenditure under financial constraints

Participants were then presented with a hypothetical scenario: they experience ‘a large, unexpected expense (e.g. tax bill, house repairs, etc.)’, meaning that there is not enough budget left to pay for their usual level of healthcare utilisation. Participants were then presented with the healthcare expenditure information set at the levels they previously described. However, their budget was now 25% less than the amount it would take to use all of these services as normal. 25% was chosen as it was considered sufficient to require decisions that would significantly affect healthcare utilisation, while low enough to ensure people were not forced to cease most of their healthcare utilisation. Participants were then asked to reduce elements of their healthcare utilisation by enough to at least align their expenditure with the new restricted budget. As the aim of the task was to shed light on which health conditions and healthcare areas people would prioritise if required to, there was no option to reduce expenditure other than healthcare (e.g. food or recreation). This design decision is grounded in evidence from behavioural economics showing that people tend to create ‘mental accounts’ for their spending and are reluctant to use money assigned to one function (e.g., groceries) to cover another (e.g., healthcare) [36]. Throughout the experiment, participants were provided with explanations and examples (See Appendix A).

After completing the choice experiment, participants were given a list of possible reasons for their prioritisation decisions and were asked to rate the degree to which each reason informed their prioritisation choices, using a Likert scale ranging from 1 (not important at all) to 5 (very important). The list included maintaining independence, symptom control, clinician advice, staying alive, workload, and ‘other (please specify)’. These options were based on documented priorities of people with multimorbidity [31, 32].

Participants were then asked how likely they would be to use alternative ways of paying for healthcare (e.g. use savings, borrow money) if they were presented with this scenario in their real lives, based on a scale of 1 (very unlikely) to 5 (very likely). These were adapted from the options in a measure of financial burden previously employed for patients with stage three colorectal cancer [33].

### Real-life experiences of financial burden

This same composite measure of financial burden was also adapted to understand participants’ real-life experience of healthcare related financial burden (Appendix A, Q7-8). This included a question asking participants how much they worry about financial problems that have resulted from the cost of their healthcare on a scale of 1 (not at all) to 7 (very much). It also included questions about whether they had ever used alternative ways of paying for healthcare (e.g. use savings, borrow money), participants could choose all options that applied to them.

Participants were then asked about the number of occasions they had not purchased medicines in the previous 12 months because of cost and the number of occasions they had not attended a healthcare professional in the previous 12 months because of cost. Twelve months was selected as the reference period to reduce the risk of generating unrealistic snapshot of patient spend, as financial shocks such as those that might force a tighter budget constraint are (by definition) experienced infrequently [35]. Longer time periods can result in inaccurate estimates of expenditure due to recall bias [35], however we focused instead on occasion frequency rather than exact costs.

### Pilot

The survey company and the researchers conducted a pilot study with 60 participants. Based on the pilot responses some minor changes were made: minor technical issues were addressed, estimated completion time was revised, the wording of questions 4 and 4b were slightly revised with more detail added to ensure understanding, and an example of healthcare-related travel expenses was added to question 3.1.1. The changes were not considered substantial and therefore the pilot participants were included in the final sample.

### Patient & public involvement (PPI)

The survey was developed in consultation with a PPI panel of people with multimorbidity. To develop the financial burden scenario, members of the panel were asked to provide examples of scenarios that are likely to cause financial burden to a large proportion of people with multimorbidity. The panel was also presented with the survey in its entirety and asked a series of questions to ensure comprehension (Appendix B, eBox 7). One of the members of the PPI panel (co-author, TH) was included as a co-researcher and contributed to conceptualisation, methodology, and reviewing and editing.

### Data analysis

A multimorbidity count was developed by combining some of the 31 conditions in the questionnaire to give 21 broader conditions (Appendix B, eBox 1), based on data availability and a previous Irish Longitudinal Study of Ageing study on multimorbidity [40]. For example, chronic lung disease and asthma were combined into chronic respiratory disease. Conditions were also classified as either a physical or mental condition using a previous TILDA analysis [41]. For conditions not covered by the previous analysis, a classification was made by an author (SS) who is a GP and academic with multimorbidity expertise.

As this study represents a first investigation of its kind in broader implications of out-of-pocket costs for multimorbidity, our focus was on descriptive statistics. Frequencies and descriptive statistics were used to describe the participant demographics and their reported real-life experience of financial burden. Descriptive statistics were used to describe the average monthly healthcare expenditure in each given area. Average absolute reduction and average percentage difference was used to describe difference in healthcare expenditure under financial constraints in the choice experiment. These descriptive statistics were analysed based on type of healthcare (e.g. prescription medicines, general practice etc.) and number of chronic conditions (one condition, two conditions and three or more conditions). The categories ‘two conditions’ and ‘three or more conditions’ were included to align with previous studies of multimorbidity and out-of-pocket healthcare expenditure [2].

To assess prioritisation between conditions, proportionate unit reduction was used. This method was adopted as no standard analysis for this type of data was identified in the literature. Only those with expenditure on two or more conditions (including multiple/other conditions) were included. Proportionate unit reduction was determined by calculating the percentage reduction for each condition and summing those percentages. This total percentage was then divided by the number of conditions (including *multiple/other conditions* as a condition). A proportionate unit reduction of a value less than 0 (e.g. -0.5) represents a de-prioritisation of the respective condition and a proportionate unit reduction of a value greater than 0 (e.g. 0.4) represents a prioritisation of the respective condition. An example of the proportionate unit reduction method for a participant can be seen in Appendix B, eBox 8. To assess whether participants generally treated conditions equally or tended to prioritise a condition, the average proportionate unit reduction for all participants’ most prioritised condition and least prioritised condition, was taken. The specific condition that was prioritised or de-prioritised varied from participant to participant. ‘Multiple/other conditions’ was not included when averaging the proportionate unit reduction for most and least prioritised conditions. This was because prioritising this category may mean that an individual is prioritising their overall health and not just ‘other conditions’ specifically.

Medians and interquartile ranges were used to analyse Likert scale questions. A conventional content analysis [42] of open ended questions 4b, 5b and 7 was conducted. These were free-text questions related to what informed participants’ prioritisation decisions, alternatives to sacrificing healthcare and participants’ real-life experiences of making sacrifices due to healthcare costs. The first stage involved one author (JL) reading and re-reading the responses. The second stage involved creating codes under which responses could be categorised. The third stage consisted of assigning data to the codes. A second author (LF) crosschecked this process independently. Discrepancies were addressed through discussion.

The representativeness of the sample was assessed by comparing it, using chi square tests, with those aged 50 years and over with one or more condition in the 2016 sample of the Irish National longitudinal Study on Ageing.

### Outlier management

All healthcare expenditure data that was greater than two standard deviations from the mean was reviewed by a health services researcher (JL) and a GP (SMS). Expenditure that JL and SMS considered unlikely to be feasible was removed. Reasons for this included allocation of excessively high costs for a particular area that is known not to incur such costs. This led to expenditure data for 10 of the 962 participants being removed. An example of an outlier that was removed was €555 for three GP visits. The same process was applied for cost-related non-adherence data and led to data for five of the 962 participants being removed (more details in Appendix E).

### Ethics statement

Ethical approval for this study was obtained from the RCSI University of Medicine and Health Sciences Research Ethics Committee (reference number: 202104018). The data was fully anonymised before being accessed by the research team. All participants provided written informed consent to participate in the study and for their responses to be used for research purposes.

## Results

### Participant characteristics

**Fig. 1:**
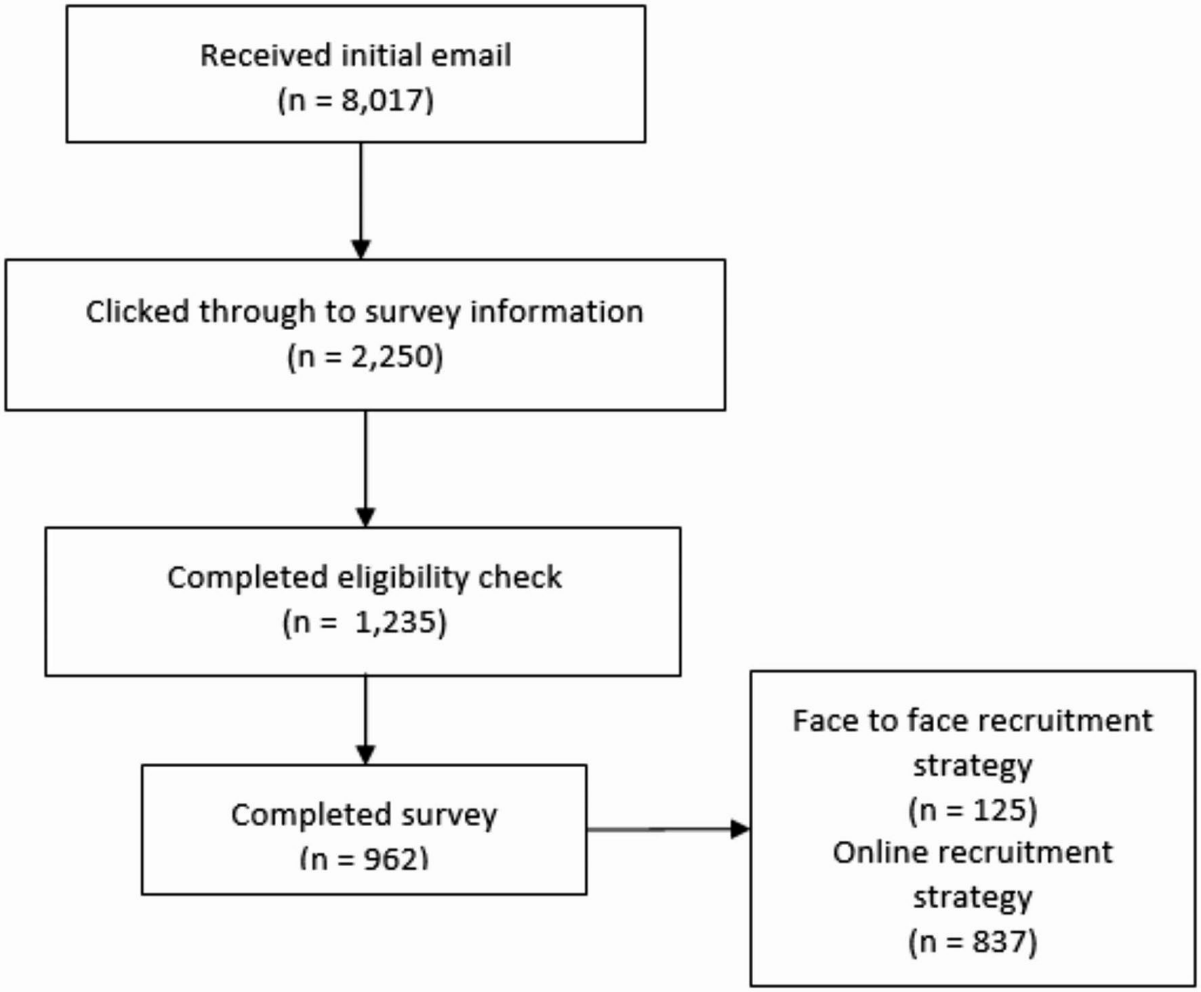
Flow chart of response rate

In total, 962 respondents completed the survey. Of these, 13.0% (*n* = 125) were recruited face-to-face to complete the online survey (further details of face-to-face recruits and online recruits can be seen in Appendix D eTable 1). A flow chart of the response rate can be seen in Fig. 1.

Overall, 54.8% (*n* = 527) of participants were female, 16.2% (*n* = 156) had a primary school education or less, 55.1% (*n* = 530) were aged between 40 and 59 years old, and the mean number of conditions was 2.4 (SD = 1.5, 95%CI: 2.3–2.5). Table 1 provides details of the sample characteristics. A breakdown of participants’ health conditions is reported in Appendix D, eTable 2. The sample was relatively representative when compared with those aged 50 years and over with one or more condition in the 2016 sample of the Irish National longitudinal Study on Ageing (the Irish National longitudinal Study on Ageing only recruited participants aged 50 years and over). However, the current sample skewed younger and had a greater proportion of males compared to the TILDA sample. Details of this comparison are in Appendix D, eTables 3 and 4.

**Table 1:** Demographic and entitlement characteristics of sample

	Overall (*n* = 962) % (*n*)	One condition (*n* = 338) % (*n*)	Multimorbidity	
			Two conditions (*n* = 265) % (*n*)	Three or more conditions (*n* = 359) % (*n*)
**Age (years)**				
40–49	22.8 (219)	31.6 (107)	18.9 (50)	17.3 (62)
50–59	32.3 (311)	32.8 (111)	38.1 (101)	27.6 (99)
60–69	29.8 (287)	26.0 (88)	30.2 (80)	33.1 (119)
70–79	11.8 (113)	8.3 (28)	11.3 (30)	15.3 (55)
80–89	2.5 (24)	1.2 (4)	1.1 (3)	4.7 (17)
90+	0.8 (8)	0.0 (0)	0.4 (1)	1.9 (7)
**Sex**				
Female	54.8 (527)	48.5 (164)	56.2 (149)	59.6 (214)
Male	45.1 (434)	51.4 (174)	43.4 (115)	40.3 (145)
Other	0.1 (1)	0.0 (0)	0.4 (1)	0.0 (0)
**Education**				
Primary/none	16.2 (156)	13.0 (44)	15.2 (40)	20.1 (72)
Secondary	36.7 (353)	36.1 (122)	37.5 (99)	36.8 (132)
Third/higher	47.0 (452)	50.9 (172)	47.3 (125)	43.2 (155)
**Location**				
Urban (5000 + people)	53.1 (511)	58.6 (198)	50.2 (133)	50.1 (180)
Rural (< 5000 people)	46.9 (451)	41.4 (140)	49.8 (132)	49.9 (179)
**Private Health Insurance**				
Yes	54.7 (526)	55.6 (188)	54.0 (143)	54.3 (195)
No	45.3 (436)	44.4 (150)	46.0 (122)	45.7 (164)
**Healthcare Entitlements**				
Medical Card	42.3 (407)	34.3 (116)	41.9 (111)	50.1 (180)
GP Visit Card	10.8 (104)	8.6 (29)	10.2 (27)	13.4 (48)
Neither	46.9 (451)	57.1 (193)	47.9 (127)	36.5 (131)

### Choice experiment: current expenditure and response to hypothetical tighter budget constraints for four healthcare areas

Participants’ median monthly healthcare expenditure was highest for ‘other healthcare (hospital visits, specialist doctors, etc.)’ at €100 (IQR: €15-€200) amongst the 473 respondents with this type of expenditure. Median monthly healthcare expenditure was lowest for medicines, at €28 (IQR: €10-€60) amongst the 709 respondents with medicine expenditure. Overall, 6.2% (*N* = 43) of those who had expenditure on medicines reached a relevant medicine payment threshold (described in Sect. 2.2) associated with their entitlements, for example the upper limit of €114 per month on medications (more details in Appendix D, eTable 6).

When participants were presented with the hypothetical tighter budget constraints, they chose to reduce other healthcare expenditure by the greatest percentage (83.0%) and medicines by the lowest percentage (28.6%). This pattern was consistent regardless of the number of chronic health conditions the participant reported. Further details broken down by number of conditions are in Table 2. Averages and standard deviations for the same variables are provided in Appendix D, eTable 5.

**Table 2:** Expenditure reductions in response to financial constraints

		Previous month’s healthcare expenditure Median (IQR)	Monthly healthcare expenditure after choices made under financial constraints Median (IQR)	Reduction in expenditure Median	Median percentage reduction in expenditure
Overall	GP	€60 (€20-€100)	€35 (€4-€60)	€25	41.7%
	Medicines	€28 (€10-€60)	€20 (€6-€46)	€8	28.6%
	Primary Care (physio, occupational therapist, psychologist)	€60 (€10-€110)	€20 (€0-€60)	€40	66.7%
	Other Healthcare (hospital visits, specialist doctors, etc.)	€100 (€15-€200)	€17 (€0-€108)	€83	83.0%
One Condition	GP	€60 (€40-€66)	€40 (€14-€60)	€20	33.3%
	Medicines	€20 (€10-€40)	€15 (€4-€30)	€5	25.0%
	Primary Care	€30 (€0-€65)	€16 (€1-€50)	€14	46.7%
	Other Healthcare	€68 (€14-€141)	€6 (€0-€70)	€62	91.2%
Two Conditions	GP	€60 (€13-€110)	€25 (€3-€65)	€35	58.3%
	Medicines	€26 (€10-€60)	€20 (€6-€47)	€6	23.1%
	Primary Care	€60 (€29-€130)	€17 (€0-€56)	€43	71.7%
	Other Healthcare	€80 (€13-€140)	€8 (€0-€75)	€72	90.0%
Three or more Conditions	GP	€60 (€15-€120)	€28 (€3-€65)	€32	53.3%
	Medicines	€39 (€12-€80)	€28 (€7-€60)	€11	28.2%
	Primary Care	€60 (€12-€120)	€27 (€0-€69)	€33	55.0%
	Other Healthcare	€130 (€20-€240)	€28 (€2-€146)	€102	78.5%

### Choice experiment: response to hypothetical tighter budget constraints for chronic conditions

Overall, 40.7% (*n* = 392) of participants reported expenditure on two or more conditions (including *multiple/other conditions* as one condition). In general, participants tended to prioritise certain conditions rather than reduce expenditure equally across conditions. The most and least prioritised conditions varied between individuals but, when considered in general terms (and multiple/other conditions is excluded), the average proportionate unit reduction for the most prioritised condition across all participants was 0.71 (SD = 0.51, 95%CI: 0.66–0.76). This represents a reduction that was 71% less than expected if expenditure across all conditions was reduced by the same proportion. For a full explanation of proportionate unit reduction refer back to the Data Analysis section of the Methods and Data and Appendix B, eBox 8.The average proportionate unit reduction for the least prioritised condition was − 0.60 (SD = 1.04, 95%CI: -0.50–0.70), which represents a reduction 60% more than expected if expenditure across all conditions was reduced by the same proportion.

### Choice experiment: influences on prioritisation choices

When asked to rate a list of six of options that influenced their prioritisation decisions, participants rated staying alive, maintaining independence, and symptom control as the most important factors, each with a median rating of 5. Doctors’ advice and treatment burden both had a median rating of 4. A more detailed breakdown can be seen in Appendix D, eTable 7. Participants were asked if there was ‘anything else’ that informed their prioritisation decisions and provided with a free-text box. Additional factors were reported by 15.6% (*n* = 150) of respondents. Of these, 46.0% (*n* = 69; 7.2% of the full sample) reported that the availability of an alternative form of care/therapy would inform their decision and 16.7% (*n* = 25; 2.6% of the full sample) said they would consider the intensity of the illness at the given point in time. Full details of alternative considerations are in Appendix D, eTable 8.

### Choice experiment: alternative cost-saving choices

**Fig. 2:**
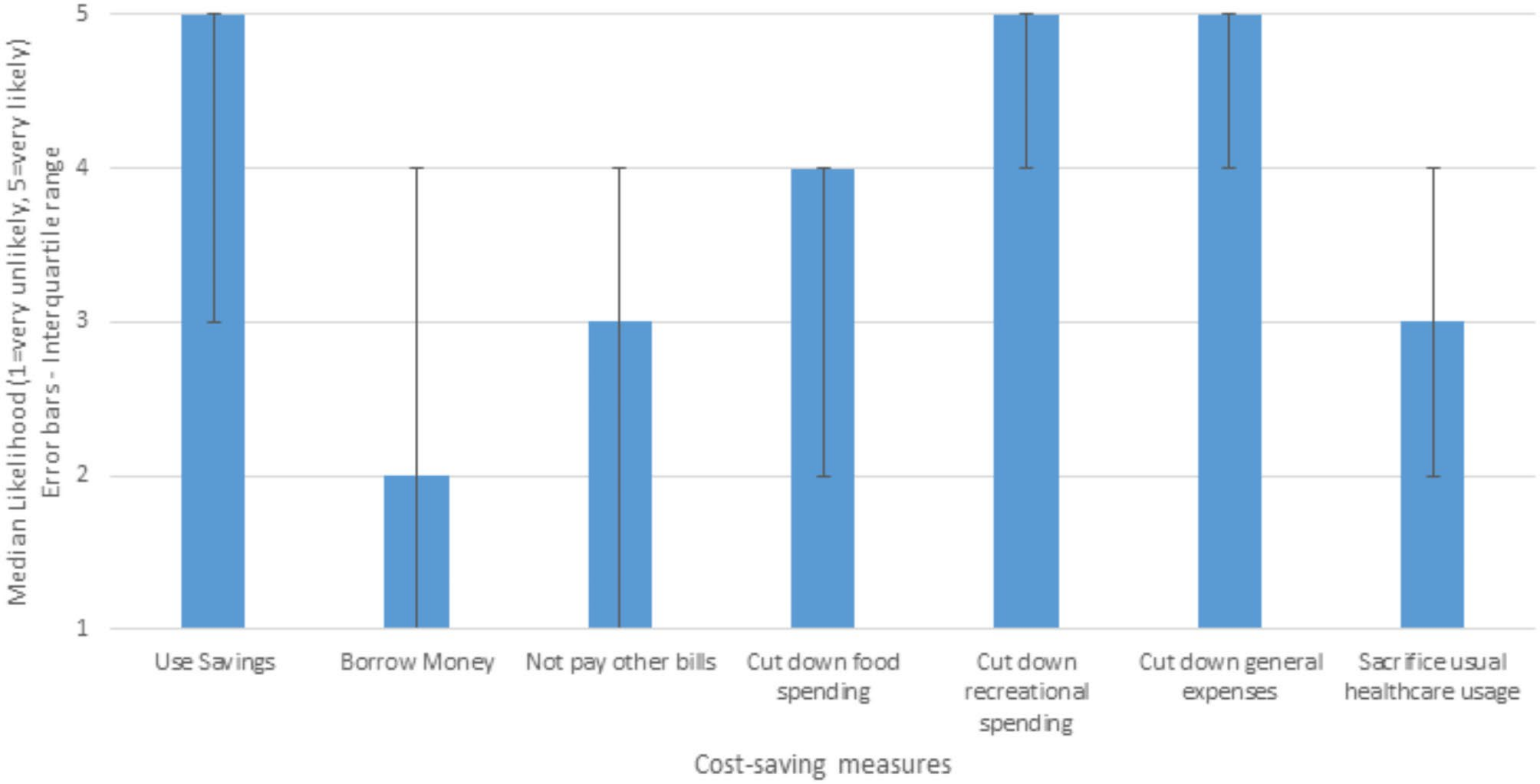
Hypothetical likelihood of cost saving measures when faced with healthcare cost challenges (*N* = 962)

Almost half (46.1%) of respondents stated that they were either likely or very likely to sacrifice parts of their usual healthcare usage if they were faced with not having enough money to access their usual level of healthcare in real life. The median response was 3 (IQR = 2–4) out of 5 (see Fig. 2). However, participants reported being more likely to use savings, cut down on recreational spending, or cut down on general non-healthcare expenses to pay for their healthcare, rather than sacrifice parts of their usual healthcare usage (Fig. 2). The distribution of responses for hypothetical cost saving decisions are in Appendix D, eTable 9.

Overall, 15.9% (*n* = 153) of participants provided free text responses when asked if there were ‘other’ alternatives they would have used to allow them to protect their usual level of healthcare. Of these, 29.4% (*n* = 45; 4.7% of the full sample) said they would increase their income (e.g. sell assets or work more), 21.5% (*n* = 33; 3.4% of the full sample) said they would change their behaviour to attempt to improve their health (e.g. through exercise, diet etc.) and avoid the need for healthcare, 4.6% (*n* = 7; 0.7% of the full sample) said they would try to negotiate with their healthcare provider, and 2.6% (*N* = 4; 0.4% of the full sample) said they would access care in another country. Full details of ‘other’ alternatives to reducing healthcare expenditure are in Appendix D eTable 10.

**Fig. 3:**
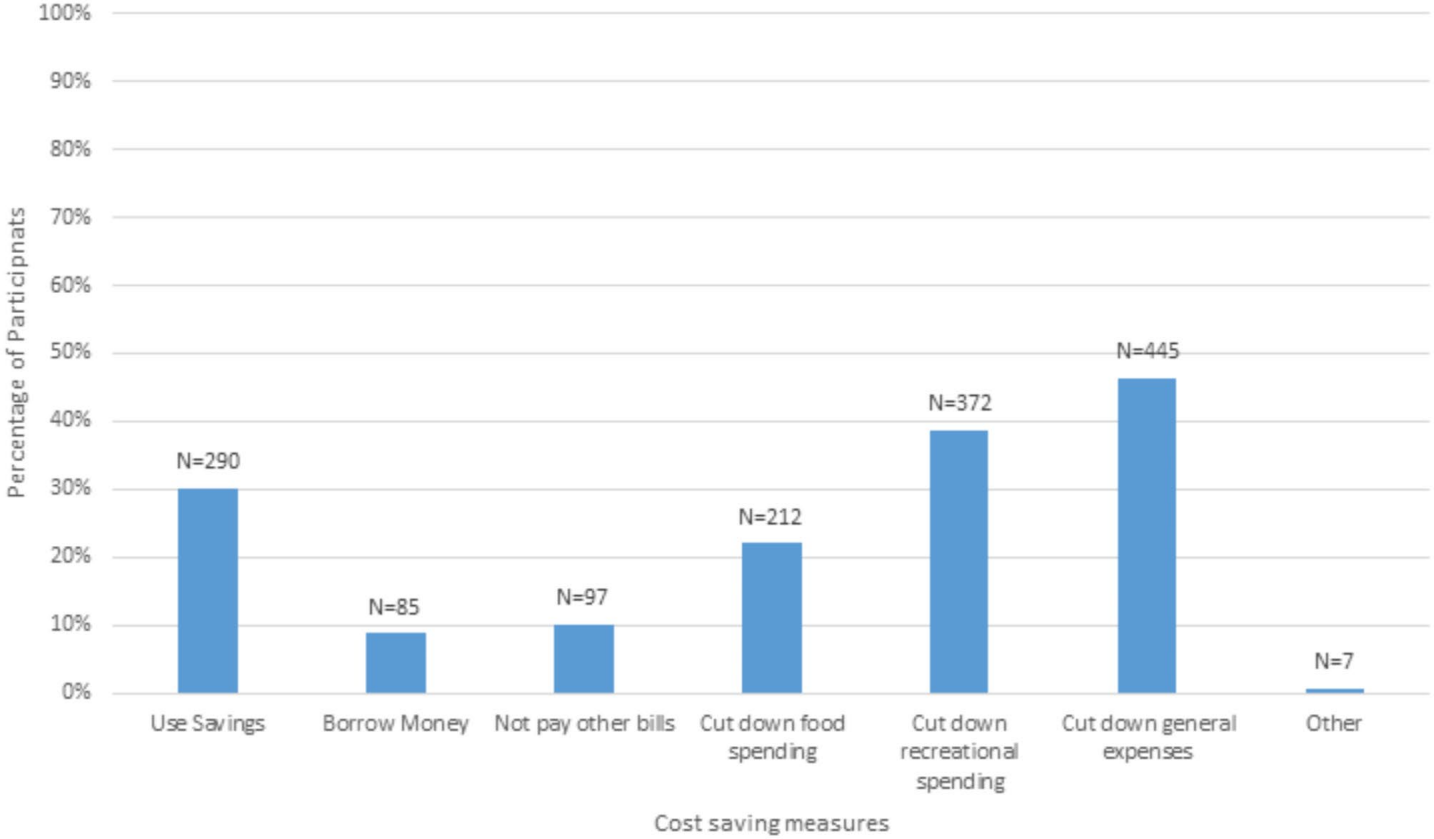
Real-life experiences of cost saving measures when faced with healthcare cost challenges (*N* = 962)

Overall, 62.3% (*n* = 599) of participants had had to use a non-healthcare cost-saving measures at some stage due to healthcare cost challenges. These real-life cost saving measures closely matched those reported for the hypothetical choice task. The most common strategy, reported by almost half (46.3%, *n* = 445) of participants, was to cut down on general expenses to pay for healthcare (Fig. 3). Cutting down on recreational spending and using savings were the next most common strategies, respectively.

Results showed some variation in reported worry about financial problems that have resulted from the cost of participant’s currently recommended healthcare treatments. The median response was 4 (IQR = 2–6) on the seven-point scale. The distribution of responses can be seen in Appendix D eTable 11.

### Real-life financial burden: cost-related non-adherence

Overall, 34.5% (*N* = 332) of participants reported some form of cost-related non-adherence to recommended healthcare in the past 12 months. The pattern of non-adherence closely matched choices in the hypothetical experiment. For healthcare visits, 31.1% (*N* = 299) of participants reported not attending a healthcare professional in the previous 12 months because of costs. Among those that had not attended, they missed these appointments an average of 2.4 (SD = 1.8, 95% CI: 2.2–2.6) times in the previous 12 months. For cost-related non-adherence to medications, 15.5% (*N* = 149) of participants reported not buying a medication in the previous 12 months because of cost. Among these, the average number of occasions that this occurred in the previous 12 months was 2.7 (SD = 2.9, 95% CI: 2.2–3.2). Table 3 provides more details of cost-related non-adherence broken down by number of conditions and healthcare entitlement status.

**Table 3:** Cost-related non-adherence/attendance in previous 12 months

	Ever not attended healthcare professional in previous 12 months due to cost % (*N*)	No. occasions Mean (SD, 95% Confidence Interval)	Ever not bought medicine in previous 12 months due to cost % (*N*)	No. occasions Mean (SD, 95% Confidence Interval)
**Medical-Card Holders**				
Overall	29.5 (120)	2.5 (2.0, 2.1–2.9)	17.7 (72)	2.9 (3.5, 2.1–3.7)
One Condition	21.6 (25)	2.4 (2.7, 1.3–3.5)	12.1 (14)	2.9 (6.1, 0.0*-6.1)
Two Conditions	27.0 (30)	2.1 (1.8, 1.5–2.7)	18.0 (20)	2.6 (2.1, 1.7–3.5)
Three + Conditions	36.1 (65)	2.7 (1.8, 2.3, 3.1)	21.1 (38)	3.1 (2.9, 2.2-4.0)
**GP Visit Card Holders**				
Overall	17.3 (18)	1.8 (1.0, 1.3–2.3)	7.7 (8)	1.7 (0.5, 1.4-2.0)
One Condition	17.2 (5)	1.8 (1.0, 0.9–2.7)	13.8 (4)	1.7 (0.6, 1.1–2.3)
Two Conditions	18.5 (5)	2.0 (1.2, 0.9–3.1)	7.4 (2)	1.5 (0.7, 0.5–2.5)
Three + Conditions	16.7 (8)	1.8 (0.9, 1.2–2.4)	4.2 (2)	2.0 (0.0, 2.0–2.0)
**Holders of neither card**				
Overall	35.7 (161)	2.4 (1.7, 2.1–2.7)	15.3 (69)	2.6 (2.2, 2.1–3.1)
One Condition	31.6 (61)	2.2 (1.3, 1.9–2.5)	14.5 (28)	2.1 (1.4, 1.6–2.6)
Two Conditions	40.2 (51)	2.5 (1.7, 2.0–3.0)	15.7 (20)	3.6 (3.3, 2.2-5.0)
Three + Conditions	37.4 (49)	2.3 (2.0, 1.7–2.9)	16.0 (21)	2.1 (1.3, 1.5–2.7)
**All Participants**				
Overall	31.1 (299)	2.4 (1.8, 2.2–2.6)	15.5 (149)	2.7 (2.9, 2.2–3.2)
One Condition	26.9 (91)	2.6 (1.8, 2.2-3.0)	13.6 (46)	2.4 (3.5, 1.4–3.4)
Two Conditions	32.5 (86)	2.4 (1.7, 2.0-2.8)	15.8 (42)	3.0 (2.8, 2.2–3.8)
Three + Conditions	34.0 (122)	2.5 (1.9, 2.2–2.8)	17.0 (61)	2.7 (2.4, 2.1–3.3)

* this has been truncated, the original statistical calculation yielded a lower bound for the 95% confidence interval below zero

## Discussion

This study used a cross sectional survey with an embedded choice experiment to systematically evaluate how people with chronic conditions, including multimorbidity, make trade-offs between their chronic conditions and between different healthcare services when faced with financial constraints. Findings show a high prevalence of cost-related non-adherence to healthcare recommendations: over a third of participants reported they had either not attended appointments or not accessed medicines due to costs in the previous 12 months. Despite prior research focusing on non-adherence to medication [13], almost twice as many participants (31%) reported previous non-attendance at a healthcare professional due to cost than non-adherence to medication for the same reason (16%).

Findings from the choice experiment, which was completed by the full sample, are consistent with these reports of lived experience with cost-related non-adherence. Almost half of participants reported that they were likely to engage in cost-related non-adherence to healthcare if faced with tighter budget constraints. Medicines were the most prioritised healthcare area, while participants prioritised ‘other healthcare (hospital visits, specialist doctors, etc.)’ the least; they reported that they would reduce their relative expenditure for ‘other healthcare’ by over three times as much as for medicines.

Among those with multimorbidity, the choice experiment showed that people tended to have a condition they were least likely to reduce expenditure for. They reduced expenditure for that condition by, on average, less than a fifth of what would be expected if they had reduced expenditure across all conditions proportionately. For their least prioritised condition, they reduced their expenditure by more than double what would be expected. The primary motivations for prioritisation were ‘staying alive’, ‘symptom control’ and ‘maintaining independence’. Whether people are likely to differentiate between these motivations is unclear, as medians for these questions were at ceiling [43]. Future research may consider implementing ranking tasks to avoid matched rationales.

Despite evidence for cost-related non-adherence to healthcare– and in particular appointments - healthcare in general was a high priority for participants. Participants stated that, if faced with tighter budget constraints, they would be more likely to use savings, cut down on recreational spending, or cut down on general expenses than reduce their healthcare expenditure. This somewhat contradicts previous research that people tend to create ‘mental accounts’ for their spending and are reluctant to use money assigned to one function to cover another [35]. These hypothetical choices mirror self-reported coping mechanisms with experienced financial burden. Importantly, however, those with chronic conditions and multimorbidity who have lower incomes and higher out-of-pocket healthcare expenditure [1] may not have the capacity to use these mechanisms.

Participants reported being less likely to borrow money to address the hypothetical financial constraints than reduce their healthcare utilisation, which may mean that there is a threshold up to which participants protect their healthcare spending if they can. Free text responses provided interesting examples of other alternatives to reducing healthcare expenditure considered by participants, such as negotiating with the healthcare provider, accessing care in another country, or increasing income through more paid work or selling personal items.

## Comparison with existing literature

Our findings that participants prioritised medication expenditure aligns with a 2017 study of older adults in Ireland with polypharmacy (prescribed five or more medications), which reported that 96% believed strongly in the necessity of their medication, with higher numbers of prescribed medicines associated with stronger beliefs [44]. Similarly, a Swedish study of ‘frail elderly patients in primary care’ found that the vast majority believed in the necessity of their medication [45]. “Doctors’ advice” was important for the majority of participants in the choice experiment when deciding which healthcare area to prioritise. It has also been reported that as functional health status decreases, belief in the necessity of medicines prescribed for multimorbidity increases, further highlighting the perceived centrality of medicines in the management of multimorbidity [46]. However, given that there is very little research examining the perceived necessity of, or beliefs about, other healthcare areas, it is difficult to put these prioritisation decisions into a wider context. In relation to prioritisation of conditions, our findings align with several qualitative studies showing that people prioritise individual conditions when engaging in medication taking behaviours [47, 48].

Despite the finding that medicines was the area that participants prioritised in response to hypothetical tighter budget constraints, about one in six participants had engaged in cost-related medication non-adherence in the previous year. Some studies have found that a small increase in costs can lead some people to reducing their medicine use, but some continue to adhere to medicines even when experiencing significant financial burden [13]. There are mixed results in the literature on whether independence, symptom control or mortality risk are more important to people when prioritising medicines [13, 49]. Our findings show that all these areas are very important for people when prioritising their conditions and their healthcare. This finding is somewhat expected given the clinical diversity inherent in multimorbidity, and the potential for people to be living with multiple co-occurring conditions which simultaneously increase their risk of mortality [50] and present symptoms which reduce quality of life and independence [51].

The proportion of participants (16%) who reported cost-related medication non-adherence in the previous year is generally higher than rates reported internationally. A study incorporating a series of cross sectional surveys of cost-related prescription medication non-adherence in the previous year among older adults in 11 high-income countries found rates of between < 3% (France, Norway, Sweden, Switzerland and the UK) and 17% (USA) [52]. However, the setting and populations differed between the choice experiment and the international surveys [52]. It also included prescription medicines only, while our study examined ‘medication you needed for your treatment.’

## Strengths and limitations

Previous studies have explored the priorities of individuals with multimorbidity using qualitative interviews [49, 53], whereas the current study combined a choice experiment with a quantitative survey among a large national sample targeting people with multimorbidity. The generalisability of findings from the choice experiment is somewhat limited, given hypothetical bias and the lack of options for participants to respond to the tighter budget constraints through mechanisms other than non-adherence. However, the strong concordance between both of our quantitative approaches gives confidence in the reliability of the findings for situations in which other mechanisms may not be possible for people. The primary hypothetical bias is that the budget constraint is fixed at 25%, which was considered sufficient to require decisions that would significantly affect healthcare utilisation, while low enough to ensure people were not forced to cease most of their healthcare utilisation. This fixed reduction allowed for comparability across participants. Nonetheless, given the greater vulnerability to financial constraints of low-income groups, a dynamic budget constraint based on participants’ income may have provided more valid results. The overall findings are somewhat generalisable as multimorbidity [2–5] and cost-related non-adherence [52] are common phenomena internationally. However, the unique healthcare entitlements system in Ireland somewhat limits the international generalisability.

A further limitation of the study is that several parts of the questionnaire, such as the healthcare utilisation and expenditure questions, were designed specifically for the study and had not been validated in other samples. That said, most questions were adapted from previous studies and all were informed by a multimorbidity PPI panel and pilot study. Also, using disease count to represent the experience of multimorbidity has limitations, for example severity of each condition and the related need for healthcare utilisation is not considered [54].

As with all studies, attrition rates pose a challenge for generalisability. The high rate of non-completion (22.1%) amongst those who started the survey is an important consideration for generating implications, as it is unclear whether non-completion is more or less likely among those who may experience cost-related non-adherence or tighter budgetary constraints. However, the rate of non-completion is not outside the range of non-completion seen in other similar online surveys [55]. The lack of data on the number of people approached in-person to complete the survey is also a limitation. The final sample showed limited representativeness when compared with those in Ireland aged 50 years and over with one or more chronic condition, as the final sample skewed younger and had a slightly greater proportion of males. The large age discrepancy limits the generalisability, as those aged over 70, who are underrepresented, likely experienced very different financial circumstances, as a result of retirement, welfare benefits and other age-specific factors. In Ireland, some areas of healthcare (e.g. medicines and hospital stays) have a threshold over which the state pays the excess. Participants at these thresholds may have been less likely to reduce expenditure in the respective area, as it may have involved making a significant change to their healthcare access. However, only a small number of participants reached these thresholds.

The responses to questions about lived experience of cost-related non-adherence referred to the previous year. Despite our efforts to reduce the influence of recall bias (e.g., by eliciting counts rather than expenditure estimates), responses are nonetheless vulnerable to it and would benefit from longitudinal monitoring of patient experiences. Another notable limitation is that any analysis of the relationship between number of chronic conditions and prioritisation decisions may be subject to confounding.

## Policy and practice

This study implies a need for policymakers to consider the impact of financial burden on treatment choices and adherence. People with multimorbidity are disproportionately represented among lower socio-economic groups and our findings show that one-in-three report cost-related non-adherence to healthcare recommendations in the previous 12 months. Participants’ reports of their lived experiences and findings from the choice experiment show that people prioritise distinct healthcare areas and conditions when faced with financial constraints, providing rational reasons such as staying alive, controlling symptoms, and maintaining independence. Non-adherence has potentially serious implications for patients and healthcare systems.

To prevent financial burden, reducing the payment barriers to accessing care is an obvious solution to cost-related non-adherence and non-attendance at a system level, given that user fees are associated with a reduction in healthcare utilisation [56]. Where this is not feasible, patients may benefit from explicit consideration of how costs might affect their adherence to recommended treatments. For example, discussion of financial costs and/or coverage related to health or healthcare in clinical practice as part of a cost-of-care conversation between patients and healthcare workers could be employed [57]. These cost-of-care conversations should be promoted, as they rarely take place despite having the potential to reduce patient costs [58] and increase adherence [59]. Consideration of referral to a welfare rights advisor or social worker could also be considered when people are experiencing financial burden [60]. These efforts could be targeted at those most vulnerable to cost-related non-adherence: those with chronic conditions, limited entitlements and of younger age [61].

## Future research

In relation to future research in this area, a study examining people’s real-life experiences of financial burden, healthcare prioritisation and cost-related non-adherence, using survey data or a healthcare database, should be prioritised. A study of this nature could provide further insights into how the decisions described here manifest in a real-world healthcare context. Also, to understand the mechanisms informing people’s prioritisation decisions in more depth, a ‘think aloud’ [62] version of this choice experiment should be prioritised, as it would offer a means of triangulating the findings of this quantitative study with qualitative insights into how and why people make choices about their healthcare expenditure. This would involve participants verbalising their thoughts as they complete the prioritisation task (prioritising healthcare under financial constraints) or retrospectively [62]. Researchers could then record, transcribe, and analyse this qualitative data to understand the assumptions, beliefs and experiences that informed participant’s decision making [62]. A future study in this area could also explore alternative approaches to budget constraints such as a dynamic budget constraint based on participants’ income (income remaining after expenditure on subsistence). Future research in this area should aim to achieve greater representation of older adults, primarily by offering non-electronic options for survey completion.

Future research should consider modelling the relationship between number of chronic conditions and healthcare prioritisation decisions under financial constraints, while adjusting for relevant variables to address confounding. Future research of the relationship between multimorbidity and healthcare prioritisation decisions under financial constraints could also consider weighted measures of multimorbidity such as the Charlson index [63], or secondary analysis of this dataset broken down by physical multimorbidity, mental multimorbidity and physical-mental multimorbidity.

## Conclusion

This study’s findings suggest that many people with multimorbidity experience cost-related non-adherence to healthcare and, when faced with tighter budget constraints, choose to prioritise their medicines. They also tend to have a condition that they have demonstrated is of greatest importance to them from a management perspective. Consideration should thus be given to reducing financial barriers to healthcare access and to facilitating healthcare workers in engaging in cost-of-care conversations with patients to explore financial costs of healthcare and healthcare coverage. More broadly, our method of combining a quantitative survey of lived experience with a choice experiment presents a novel method for exploring the impact of financial burden on patients. Overall, researchers and clinicians need to take greater consideration of the harmful effects of high healthcare costs experienced by people with multimorbidity, which can lead to healthcare choices that can have negative long-term effects on health.

## Electronic supplementary material

Below is the link to the electronic supplementary material.

Supplementary Material 1

Supplementary Material 2

Supplementary Material 3

Supplementary Material 4

Supplementary Material 5

## Data Availability

Researchers interested in using the data associated with this study may access the data for free from the following sites: Irish Social Science Data Archive (ISSDA) at University College Dublin https://www.ucd.ie/issda/data/choiceexperiment/.
