# Supplementary material for: How do people with multimorbidity prioritise healthcare when faced with tighter financial constraints? A national survey with a choice experiment component
Source: BMC Prim Care. 2025 Feb 27;26:57. doi: 10.1186/s12875-025-02738-9 (PMC11866811; doi:10.1186/s12875-025-02738-9)
Supplement: Supplementary file 1 — Supplementary Material 1 [file 12875_2025_2738_MOESM1_ESM.docx]

We would firstly like to start by asking you about your health conditions

**Question 1.1.1**

Has a doctor ever told you that you have one of the following conditions? MULTICODE.

| **Respiratory** | |
| --- | --- |
| Chronic lung disease such as chronic bronchitis or emphysema |  |
| Asthma |  |
| **Musculoskeletal/bone disease** | |
| Arthritis (including osteoarthritis, or rheumatism) |  |
| Osteoporosis, sometimes called thin or brittle bones |  |
| **Cancer** | |
| Cancer or a malignant tumour (including leukaemia or lymphoma but excluding minor skin cancers) |  |
| **Neurological** | |
| Parkinson's disease |  |
| Alzheimer's disease |  |
| Dementia, organic brain syndrome, senility |  |
| Serious memory impairment |  |
| **Mental Health** | |
| Any emotional, nervous or psychiatric problems, such as depression or anxiety |  |
| Alcohol or substance abuse |  |
| **Gastrointestinal (Gastric Conditions)** | |
| Stomach ulcers |  |
| Cirrhosis, or serious liver damage |  |
| **Diabetes or high blood sugar (Endocrine)** | |
| Diabetes or high blood sugar |  |
| Thyroid disease |  |
| **Cardiovascular (Heart)** | |
| High blood pressure or hypertension |  |
| Angina |  |
| A heart attack (including myocardial infarction or coronary thrombosis) |  |
| Congestive heart failure (heart failure) |  |
| High cholesterol |  |
| A heart murmur |  |
| An abnormal heart rhythm |  |
| Any other heart trouble (specify)………………………… |  |
| **Vascular/Veins** | |
| Varicose Ulcers (an ulcer due to varicose veins) |  |
| A stroke (cerebral vascular disease) |  |
| Ministroke or TIA (transient ischemic attack) |  |
| Peripheral vascular disease |  |
| **Eye disease** | |
| Cataracts |  |
| Glaucoma |  |
| Blurred vision or no vision (Age related macular degeneration) |  |
| Other eye disease (specify) |  |
| None of these (IF SELECTED, CLOSE SURVEY) |  |

Could you please provide a few details about yourself? This will help us understand how costs may vary depending on a participant’s individual circumstances.

**Question 2.1.1**

What is your age in years?

18-24………………………………………………… 1

25-29………………………………………………… 2

30-34………………………………………………… 3

35-39………………………………………………… 4

40-44 5

45-49 6

50-54 7

55-59 8

60-64 9

65-69 10

70-74 11

75-79 12

80-84 13

85-89 14

90+ 15

**Question 2.2.1**

Are you?

Male 1

Female 2

Other 3

Prefer not to say 4

**Question 2.3.1**

What is your marital status?

Single 1

Married or cohabiting 2

Separated 3

Divorced 4

Widowed 5

Other (Specify) 6

**Question 2.4.1**

Which county do you live in?

| [Carlow](https://www.geni.com/projects/County-Galway-Ireland-Main-Page/17687) | 1 |
| --- | --- |
| [Cavan](https://www.geni.com/projects/County-Sligo-Ireland-Main-Page/17720) | 2 |
| [Clare](https://www.geni.com/projects/County-Wexford-Ireland-Main-Page/17772) | 3 |
| [Cork](https://www.geni.com/projects/County-Wicklow-Ireland-Main-Page/17774) | 4 |
| [Donegal](https://www.geni.com/projects/County-Louth-Ireland-Main-Page/17708) | 5 |
| [Dublin](https://www.geni.com/projects/County-Dublin-Ireland-Main-Page/17680) | 6 |
| [Galway](https://www.geni.com/projects/County-Clare-Ireland-Main-Page/17674) | 7 |
| [Kerry](https://www.geni.com/projects/County-Tipperary-Ireland-Main-Page/17765) | 8 |
| [Kildare](https://www.geni.com/projects/County-Donegal-Ireland-Main-Page/17678) | 9 |
| [Kilkenny](https://www.geni.com/projects/County-Roscommon-Ireland-Main-Page/17718) | 10 |
| [Laois](https://www.geni.com/projects/County-Laois-Queens-Irelend-Main-Page/17698) | 11 |
| [Leitrim](https://www.geni.com/projects/County-Kerry-Ireland-Main-Page/17692) | 12 |
| [Limerick](https://www.geni.com/projects/County-Kilkenny-Ireland-Main-Page/17696) | 13 |
| [Longford](https://www.geni.com/projects/County-Longford-Ireland-Main-Page/17705) | 14 |
| [Louth](https://www.geni.com/projects/County-Leitrim-Ireland-Main-Page/17700) | 15 |
| [Mayo](https://www.geni.com/projects/County-Cork-Ireland-Main-Page/17676) | 16 |
| [Meath](https://www.geni.com/projects/County-Kildare-Ireland-Main-Page/17694) | 17 |
| [Monaghan](https://www.geni.com/projects/County-Waterford-Ireland-Main-Page/17767) | 18 |
| [Offaly](https://www.geni.com/projects/County-Offaly-Kings-Ireland-Main-Page/17716) | 19 |
| [Roscommon](https://www.geni.com/projects/County-Westmeath-Ireland-Main-Page/17769) | 20 |
| [Sligo](https://www.geni.com/projects/County-Meath-Ireland-Main-Page/17712) | 21 |
| [Tipperary](https://www.geni.com/projects/County-Monaghan-Ireland-Main-Page/17714) | 22 |
| [Waterford](https://www.geni.com/projects/County-Cavan-Ireland-Main-Page/17672) | 23 |
| [Westmeath](https://www.geni.com/projects/County-Mayo-Ireland-Main-Page/17710) | 24 |
| [Wexford](https://www.geni.com/projects/County-Limerick-Ireland-Main-Page/17703) | 25 |
| [Wicklow](https://www.geni.com/projects/County-Carlow-Ireland-Main-Page/17656) | 26 |

**Question 2.4.3**

Which of the following best describes the area in [COUNTY] in which you live…

| A City |
| --- |
| A large town (5000+ population) |
| A small town (1,500 – 4,999 population) |
| A rural area |

**Question 2.5.1**

Do you have a full medical card?

Yes 1

No 2

**Question 2.5.2**

Do you have a GP visit card?

Yes 1

No 2

**Question 2.5.3**

Do you have private health insurance?

Yes 1

No 2

**Question 2.5.5**

Do you have travel card that entitles you to free access to public transport?

Yes 1

No 2

**Question 2.6.1**

And before continuing with this survey, please review the categories below. Then on the next screen where we ask you 'type of occupation' please choose from the list the category that applies to the chief wage earner in your household.

- **Higher managerial / professional / administrative**
  *e.g. Established doctor, Chartered Accounted, Architect, Solicitor, Board Director in a large organisation (200+ employees,top level civil servant/public service employee)*
- **Intermediate managerial / professional / administrative**
  *e.g. Newly qualified (under 3 years) Doctor or Solicitor, Board director of a small organisation, middle manager in a large organisation, principle officer in civil service/local government*
- **Supervisory or clerical; junior manager / professional / administrative**
  *e.g. Office worker with no staff, Student Doctor, Foreman with 25+ employees, office based or travelling salesperson etc*
- **Skilled manual worker**
  *e.g. Skilled Bricklayer, Carpenter, Plumber, Painter, Bus/ Ambulance Driver, HGV driver, AA patrolman, pub/bar worker, etc*
- **Semi-skilled/unskilled manual worker**
  *e.g Retail assistants, construction labourers, cleaners, waiting staff etc*
- **Casual worker - not in permanent employment**
  *Anyone working in Contract employment e.g. Temp Staff for maternity, sickness or holiday cover*
- **Full-time carer of other household member**
  *Carers of sick, disabled, elderly or infirm relatives on a full time basis*
- **Full-time farmer**
  *Anyone who's main income comes from farming, through ownership or lease of a farm*
- **Retired and living on state pension**
  *Retired, Recieving state pension and no other income*
- **Retired with other income**
  *Retired, with private pension or living off investments etc*
- **Student**
  *Full-time student in second level, a University, I.T. or post leaving certificate course*
- **Unemployed/not working due to long-term sickness**
  *Between jobs/looking for work, unable to work due to long-term sickness or disability*
- **Working in the home**
  *Stay at home mum, housewife etc*

**Question 2.7.1**

What is the highest level of education (full-time or part-time) which you have completed to date?

| No formal education or training |
| --- |
| Primary education |
| Lower secondary education (Junior/Inter/Group Cert, O levels/GCSEs, NCVA Foundation Cert., Basic Skills Training Cert. or equivalent) |
| Upper secondary education (Leaving Cert. (including Applied and Vocational programmes), ‘A’ Levels, NCVA Level 1 Cert., Teagasc Cert./Diploma or equivalent) |
| Technical or Vocational (Completed Apprenticeship, NCVA Level 2/3 FETAC Level 4/5 Cert., NCVA Level 2/3 Cert., Teagasc Cert./Diploma or equivalent) |
| Both Upper Secondary and Technical or Vocational Qualification |
| Non-Degree (National Certificate, Diploma NCEA/Institute of Technology or equivalent, or Nursing Diploma) |
| Primary Degree (Third Level Bachelor Degree) |
| Professional Qualification (of Degree status at least) |
| Both a Degree and a Professional qualification |
| Postgraduate Cert. or Diploma |
| Postgraduate Degree (Masters) |
| Doctorate (Ph.D) or higher |

**Question 2.8.1**

We understand that it is difficult to give an exact figure for household income but we would like you to estimate which grouping matches your total ***net*** household income in the last year (after any deductions for tax, social insurance (PRSI) or pension and health contributions, union dues and so on). This includes income from all sources (regular overtime, commission, tips etc.) and from all members of the household.

Per Year

Under €12,000 1

€12,001 to €18,000 2

€18,001 to under €24,000 2

€24,001 to under €30,000 3

€30,001 to under €42,000 4

€42,001 to under €48,000 5

€48,001 to under €54,000 6

€54,001 to under €60,000 6

€60,001 to under €78,000 7

€78,001 to under €96,000 8

€96,001 or more 9

**Question 2.9.1**

How many adults (16 years or older) are there in your household?

Number of adults ___

**Question 2.9.2**

How many children (15 years or younger) are there in your household?

Number of children ___

- In the next section, you will be asked to estimate your costs of healthcare over the ***last month***. This will be broken down into the following categories:
  - GP Visits
  - Medicines
  - Primary Care (e.g physio, occupational therapists, psychologists, etc) Visits
  - Other Healthcare (e.g hospital appointments, specialist doctors, etc) Visits
- Please note that this estimate is to ***include travel expenses*** (e.g. parking and fuel for journeys to pharmacy, GP or hospital).
- The exercise will be in a grid form which will list out the various healthcare categories listed above (e.g. GP visits, medicine, etc.), alongside the ***chronic conditions you listed*** in the first question of the survey. So, for example, if you listed Diabetes and Depression as chronic conditions you are experiencing, then these will appear as separate conditions under the healthcare category which you will then fill in the costs for.
- A ‘multiple/other illnesses’ option will also be included as a separate category to allow for ***healthcare usage that applies to more than one illness***, for example, if we use the same example of a person having diabetes and depression, when that ***person visits a GP they may do so in order to discuss both their diabetes and their depression***. This can also apply to scenarios where you access healthcare or medication for something that is not directly related to one of your illnesses (e.g. medicines for the flu).
- The total costs will be shown at the top of the grid if you need to check this during the exercise.
- We will now show you an example of how to fill in your healthcare costs correctly.


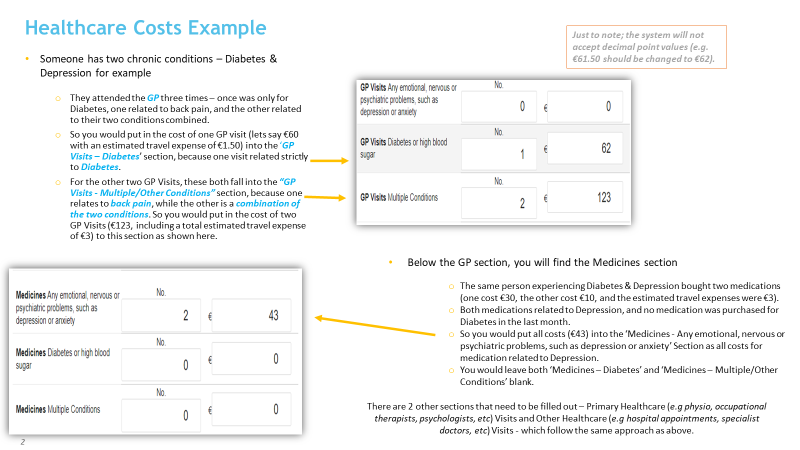


**ASK Q3.1.1 FOR EVERY CONDITION SELECTED FROM Q1.1.1 IN EACH SUBJECT AREA (e.g GP Visits, Medicines, Primary Care Visits, Other Healthcare Visits)**

**Question 3.1.1**

In the boxes below, please estimate your use of healthcare for the last month and also how much each aspect of this healthcare use cost you (including travel expenses such as parking and fuel for journeys to pharmacy, GP or hospital)

COST OF €0 ALLOWED

| **Healthcare Service** | **Number** | **Total Cost in Last Month** |
| --- | --- | --- |
| ***GP Visits*** |  |  |
| GP Visit – **CODE FROM Q1.1.1** (e.g Diabetes) |  |  |
| GP Visit – **CODE FROM Q1.1.1** (e.g Depression) |  |  |
| GP Visit – Multiple/other illnesses |  |  |
| ***Medicines*** |  |  |
| Medicines – **CODE FROM Q1.1.1** (e.g Diabetes) |  |  |
| Medicines – **CODE FROM Q1.1.1** (e.g Depression) |  |  |
| Medicines – Multiple/other illnesses |  |  |
| ***Primary Care (physio, occupational therapist, psychologist) Visits*** |  |  |
| Primary Care Visits – **CODE FROM Q1.1.1** (e.g Diabetes) |  |  |
| Primary Care Visits – **CODE FROM Q1.1.1** (e.g Depression) |  |  |
| Primary Care Visits – Multiple/other illnesses |  |  |
| ***Other Healthcare (hospital visits, specialist doctors, etc) Visits*** |  |  |
| Other Healthcare Visits – **CODE FROM Q1.1.1** (e.g Diabetes) |  |  |
| Other Healthcare Visits – **CODE FROM Q1.1.1** (e.g Depression) |  |  |
| Other Healthcare Visits – Multiple/other illnesses |  |  |
| **Total Cost in Last Month** |  |  |

**ASK Q3.1.2 FOR EVERY CONDITION SELECTED FROM Q1.1.1. INCLUDE A “MULTIPLE/OTHER ILLNESSES” CATEGORY FOR ALL RESPONDENTS.**

Q.3.1.2

- Let’s imagine a ***hypothetical scenario***;  you’re faced with a ***large unexpected expense*** (e.g. a tax bill, house repairs etc.) and there isn’t enough left over for your usual monthly healthcare usage. Given the situation, you will need to ***reduce your usual level of monthly healthcare spend by 25%***.
- In order to do this, you need to make ***some trade-offs*** below. Looking at the number of times in the past month that you bought/visited various healthcare professionals/medicines, you will need to reduce your budget to get below the ***€Y*** (reduced) monthly amount.
- Please ***reduce some areas of your usual healthcare usage*** in order to reduce your spend to €Y per month. ***It doesn’t have to precisely match the €Y amount; it can be lower if needed.***
- The exercise will be presented in the same format as the healthcare costs question (grid form) and will show you the ***reduced budget*** that you need to reach and your ***current spend*** to help guide you. Please note that we are ***focused solely on the total costs*** and not the quantity of visits or medications.
- Therefore, it is important that you follow ***real-life examples of costs*** when reducing your healthcare spend – so for example, you ***should not reduce your total cost for GP visits by only €5*** as this is not realistic. Stick to actual costs and reduce your budget with this in mind.


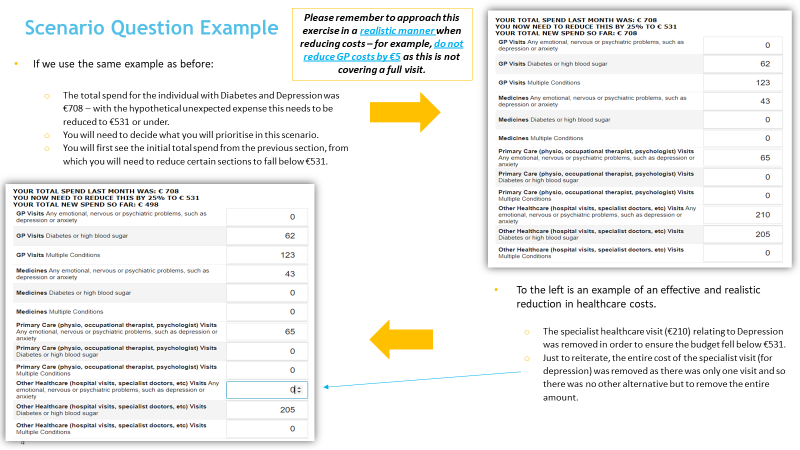


**YOUR TOTAL SPEND LAST MONTH WAS: €X**

**YOU NOW NEED TO REDUCE THIS BY 25% TO €Y**

**YOUR CURRENT SPEND IS €_____ (CODE TO ENSURE CANNOT GO ABOVE Y)**

| **Healthcare Service** | **Total Cost in Last Month** |
| --- | --- |
| ***GP Visits*** |  |
| GP Visit – **CODE FROM Q1.1.1** (e.g Diabetes) |  |
| GP Visit – **CODE FROM Q1.1.1** (e.g Depression) |  |
| GP Visit – Multiple/other illnesses |  |
| ***Medicines*** |  |
| Medicines – **CODE FROM Q1.1.1** (e.g Diabetes) |  |
| Medicines – **CODE FROM Q1.1.1** (e.g Depression) |  |
| Medicines – Multiple/other illnesses |  |
| ***Primary Care (physio, occupational therapist, psychologist) Visits*** |  |
| Primary Care Visits – **CODE FROM Q1.1.1** (e.g Diabetes) |  |
| Primary Care Visits – **CODE FROM Q1.1.1** (e.g Depression) |  |
| Primary Care Visits – Multiple/other illnesses |  |
| ***Other Healthcare (hospital visits, specialist doctors, etc) Visits*** |  |
| Other Healthcare Visits – **CODE FROM Q1.1.1** (e.g Diabetes) |  |
| Other Healthcare Visits – **CODE FROM Q1.1.1** (e.g Depression) |  |
| Other Healthcare Visits – Multiple/other illnesses |  |
| **Total Cost in Last Month** |  |

At this point, we would like to ask about why you made the decisions that you made.

Q.4 In the previous set of questions, you were asked to prioritise some services and sacrifice others.

When considering what should be prioritised, how important are/were the following areas in informing your decisions?

Please rate each on a scale from 1-5, from not at all important to very important.

**(Participants are presented with each option of Q.4 individually and the order in which each respondent sees the option is random)**

- **Maintaining your independence (ensuring you can take care of yourself, minimising the need for help from others)**

Not important Not Neither important Important Very

at all Important nor not important important

1 2 3 4 5

- **Keeping your symptoms under control**

Not important Not Neither important Important Very

at all Important nor not important important

1 2 3 4 5

- **Advice from your doctors**

Not important Not Neither important Important Very

at all Important nor not important important

1 2 3 4 5

- **Staying alive**

Not important Not Neither important Important Very

at all Important nor not important important

1 2 3 4 5

- **The work involved in looking after your health (examples of work include travelling long distances for appointments or taking medications at inconvenient times)**

Not important Not Neither important Important Very

at all Important nor not important important

1 2 3 4 5

Q4b Is there anything else that would inform your decision making when prioritising services? If so please provide details in the box below:

|  |
| --- |

Q5. If you were presented with this scenario in your real life (not having enough money to access your usual level of healthcare), do you think you would do any of the following to make sure you can continue to use your usual level of healthcare?

Please rank on a scale of 1-5 from very unlikely to very likely.

**ROTATE ORDER.**

**Use your savings**

Very Unlikely Unlikely Neither unlikely nor likely Likely Very Likely

1 2 3 4 5

**Borrow money or take out a loan**

Very Unlikely Unlikely Neither unlikely nor likely Likely Very Likely

1 2 3 4 5

**Not make payments on non-healthcare bills**

Very Unlikely Unlikely Neither unlikely nor likely Likely Very Likely

1 2 3 4 5

**Cut down on spending for food**

Very Unlikely Unlikely Neither unlikely nor likely Likely Very Likely

1 2 3 4 5

**Cut down on spending on recreational activities**

Very Unlikely Unlikely Neither unlikely nor likely Likely Very Likely

1 2 3 4 5

**Cut down on expenses in general**

Very Unlikely Unlikely Neither unlikely nor likely Likely Very Likely

1 2 3 4 5

Q5b Is there anything else you would do to make sure you can continue to use your usual level of healthcare? If so please provide details in the box below:

|  |
| --- |

Q6 If you were presented with this scenario your real life (not having enough money to access your usual level of healthcare), do you think you would sacrifice parts of your usual healthcare usage?

Please rank on a scale of 1-5 from very unlikely to very likely

Very Unlikely Unlikely Neither unlikely nor likely Likely Very Likely

1 2 3 4 5

I’d now like to ask about your real life experience of financial issues.:

Q7 Please answer yes or no to the following questions. As a result of the cost of your currently recommended healthcare treatments have you ever:

had to use savings 1

had to borrow money or take out a loan 2

not made payments on non-healthcare bills 3

cut down on spending for food 4

cut down on spending on recreational activities 5

cut down on expenses in general.....................................6

Other sacrifices (please specify) .....................................7

None of the above 8

Q8 On a scale of 1-7 how much do you worry about financial problems that have resulted from the cost of your currently recommended healthcare treatments?

Not at all Very much

1 2 3 4 5 6 7

Q9 In the last twelve months, have you ever not attended a healthcare professional when you thought you needed to, because of the cost of the visit (or the costs associated with the visit such as travel, taking time off work etc.)?

Yes/no Number of Occasions _____

Q11 In the last twelve months, have you ever not purchased medication you needed for your treatment because of the cost?

Yes/no Number of Occasions _____

Finally, we would like to ask about your general health.

Q12 Would say your health is..

1. Excellent

2. Very Good

3. Good

4. Fair

5. Poor
