# Supplementary material for: How do people with multimorbidity prioritise healthcare when faced with tighter financial constraints? A national survey with a choice experiment component
Source: BMC Prim Care. 2025 Feb 27;26:57. doi: 10.1186/s12875-025-02738-9 (PMC11866811; doi:10.1186/s12875-025-02738-9)
Supplement: Supplementary file 2 — Supplementary Material 2 [file 12875_2025_2738_MOESM2_ESM.docx]

**eBox 1 Conditions included in multimorbidity analysis (partly based on Ryan and colleagues)**

| 1. Cardiac Condition  - Angina - Heart Attack - Congestive Heart Failure - Heart Murmur - Abnormal heart rhythm - Any heart trouble  1. Vascular disease  - Stroke - TIA - Peripheral vascular disease  1. HTN 2. Diabetes 3. High Cholesterol 4. Chronic respiratory disease  - Chronic lung disease - Asthma  1. Liver disease  - Alcohol abuse - Cirrhosis  1. Eye disease  - Cataracts - Glaucoma - Age related macular degeneration - Other eye disease  1. Cognitive Impairment  - Alzheimer’s   - Dementia   - Serious cognitive impairment  1. Arthritis 2. Osteoporosis 3. Cancer 4. Parkinson’s disease 5. Emotional /psychological condition including anxiety and depression 6. Gastrointestinal conditions including Stomach Ulcers 7. Varicose veins including varicose ulcers 8. Thyroid Problems |
| --- |

**eBox 2 Initial Invitation Email (Online Only Participants)**

| Dear,  We are inviting many hundreds of people from around Ireland to contribute to this research about decision making when it comes to healthcare. Specifically, we are interested in how people with chronic health issues prioritise their healthcare.  Even if making decisions with regard to health is not currently a priority for you day-to-day, we’re still interested in hearing your opinion. We are seeking responses from a broad cross-section of people to best reflect views and experience in Irish society.  This national research is being conducted by a team of researchers based in the REDACTED in conjunction with REDACTED. You are eligible to participate if you are forty years of age or older and a doctor has ever told you that you have a chronic medical condition (health problems that require ongoing management).  The main stage of the survey will take 25-30 minutes to complete. Participation is voluntary, so you can choose to withdraw at any time by closing your browser and not returning to the survey. Only fully completed survey responses will be included.  Those who qualify and complete the survey will receive 1,000 points, which will be added after the conclusion of the research period.   Please click below to find out more about this research and decide whether or not you would like to participate.  [Click Here to Start the Survey](https://eur03.safelinks.protection.outlook.com/?url=http%3A%2F%2Fprod01.p1central.com%2FPlatform1Landing%2FPages%2FLanding%2FTestLink.aspx%3FPARAMS%3DTDRsbTQ2bHBSbUdiYzNhRGM5ODVYMDZvcVRhVHBRbHB3NUtmYXdUSXFDc1NCMUd4N211M05MUUpzSTdtclVabDdPSHRoWDdnZWJ1OVZDWjVZNUFQeUhtY3RSSVpSNmdsL2N2TE1LL3ZNUzA90&data=04%7C01%7Cinfo%40bandasurvey.ie%7Cdc8cad98dd7745a1144c08d9f876b2cd%7C9b798812dfb14547bbe6b97c811e28f6%7C0%7C1%7C637814010961058957%7CUnknown%7CTWFpbGZsb3d8eyJWIjoiMC4wLjAwMDAiLCJQIjoiV2luMzIiLCJBTiI6Ik1haWwiLCJXVCI6Mn0%3D%7C3000&sdata=LnzXrqtvy8WuG%2Bu8o5BkWt6R%2BBVZozxJy34%2F1OI3SFc%3D&reserved=0) |
| --- |

**eBox 3 Example of Initial Invitation Email (Face-to-face recruits)**

| Dear _,  Many thanks again for going through the initial questionnaire with our interviewer Bernie and agreeing to take part in this research on behalf of the REDACTED.  Please click below to complete the survey  Start Survey  Many thanks in advance, |
| --- |

**eBox 4 Reminder email**

| Dear ,  A brief reminder of this survey invitation where we are inviting many hundreds of people from around Ireland to contribute to this research about decision making when it comes to healthcare. Specifically, we are interested in how people with chronic health issues prioritise their healthcare.  Even if making decisions with regard to health is not currently a priority for you day-to-day, we’re still interested in hearing your opinion. We are seeking responses from a broad cross-section of people to best reflect views and experience in Irish society.  This national research is being conducted by a team of researchers based in the REDACTED in conjunction with REDACTED. You are eligible to participate if you are forty years of age or older and a doctor has ever told you that you have a chronic medical condition (health problems that require ongoing management).  The main stage of the survey will take 25-30 minutes to complete. Participation is voluntary, so you can choose to withdraw at any time by closing your browser and not returning to the survey. Only fully completed survey responses will be included.  Those who qualify and complete the survey will receive 1,000 points, which will be added after the conclusion of the research period.   Please click below to find out more about this research and decide whether or not you would like to participate.  [Click Here to Start the Survey](https://eur03.safelinks.protection.outlook.com/?url=http%3A%2F%2Fprod01.p1central.com%2FPlatform1Landing%2FPages%2FLanding%2FTestLink.aspx%3FPARAMS%3DTDRsbTQ2bHBSbUdiYzNhRGM5ODVYMDZvcVRhVHBRbHB3NUtmYXdUSXFDc1NCMUd4N211M05MUUpzSTdtclVabDdPSHRoWDdnZWJ1OVZDWjVZNUFQeUhtY3RSSVpSNmdsL2N2TE1LL3ZNUzA90&data=04%7C01%7Cinfo%40bandasurvey.ie%7C320d5afe42a54fd0559708d9f8776f79%7C9b798812dfb14547bbe6b97c811e28f6%7C0%7C1%7C637814014137077778%7CUnknown%7CTWFpbGZsb3d8eyJWIjoiMC4wLjAwMDAiLCJQIjoiV2luMzIiLCJBTiI6Ik1haWwiLCJXVCI6Mn0%3D%7C3000&sdata=d8r%2FyJnVdvGqdCw%2FRaDvfP70baMwyYZ3FAJUmM8luSs%3D&reserved=0) |
| --- |

**eBox 5. Face to face to online recruitment details**

| In order to address the sampling bias arising from the online-only sampling strategy, the survey company sourced 13% of participants through a face-to-face to online recruitment process. This involved sampling a nationally representative sample of Electoral Division’s across Ireland. Each recruiter will be tasked with recruiting respondents randomly from households within the Electoral Division who are 40 years of age or over with at least one condition and who are willing to take part in the online survey. Recruiters will go to the doors of randomly chosen households, explain that they are a recruiter for Behaviour & Attitudes, seeking to recruit people aged 40 or over with one or more chronic health conditions.  A total of 49 interviewers worked across Ireland to secure face-to-face recruitment for self-completion online. To start, Electoral Divisions (EDs) locations were selected randomly from the CSO ED list across Dublin, Rest of Leinster, Munster, and Conn/Ulster as starting points, and followed a random route procedure, skipping 3 houses in urban areas and 1 house in rural areas. A random route procedure is a form of non-probability sampling, where sampling points are selected at random, known as the starting point, and interviewers are given instructions on random walking rules from this point (e.g. which direction to walk in initially, how many houses to skip, what side of the road to walk on, which roads to follow on junctions, etc.). The sample was not strictly prescriptive with some room for flexibility needed due to the difficulties sourcing suitable candidates. This difficulty surrounded internet access within the older population, with many not having adequate access or skills to complete this lengthy survey.  A number of demographic questions (for example, they will be asked their age and how many chronic conditions they have) and questions about the availability of broadband and desktop/pc were asked to identify whether the respondent will be able to complete the online survey. If they qualify, they were given the study information leaflet to explain the study and asked if they are willing to take part.  Those that agree to take part then had their contact details recorded, a support letter will then be handed to them on how they will conduct the survey online. The survey company used the personal details to send them the survey link and provided them a helpline in case they have any issues with the process. There was an explanation on how they will receive their €10 incentive as well.  A demographic break down of the face to face recruits and the online only recruits can be seen in Appendix B eTable 12. |
| --- |

**eBox 6 Support Letter (Face-to-face sourcing for online)**

| Thank you for agreeing to take part in the online survey on attitudes and decision-making regarding healthcare and the costs involved. This is an online survey which will be conducted by REDACTED on behalf of REDACTED.  You will receive an email invitation from REDACTED to the email address that you provided to us.  This email invitation will contain your link to the online survey.  Once you click on the survey link, you will be brought to an introductory web page which will outline what is involved and how to take part.  You can reply to the invitation email to contact the research team if you have any queries.  Everything you need to know will be presented on screen.  In overview, we will ask you some initial questions to confirm that the research is relevant to you and also provide a recap of all of the information about the research so that you can ultimately decide if you are happy to take part.  Navigation through the questionnaire is very straightforward. A Next Question’ button will be available at each question to progress.  If you need to take a break, you can close the survey and come back to it another time.  Click ‘Next Question’ to progress (this will be the case for all pages of the survey). A number of screening questions will then appear to ensure that you fit the criteria set out for the research. We will need to repeat these to ensure that all who take part in the survey fit the criteria.  Once you get through this section, an introductory webpage with some information on the study will be shown to you. Click ‘Next question’ to progress to the consent page. You need to read this carefully and if you are comfortable to proceed, please click ‘Next question’.  The main section of the survey will then begin. This should take around 25-30 minutes in total to complete. Please read each question carefully and answer as accurately as you can.  Once completed, you will receive a follow up email from REDACTED on behalf of REDACTED with information on the €10 incentive. |
| --- |

**eBox 7. PPI Panel Questions**

| Did you have any issues understanding what was asked of you?  What would you find difficult about answering the questions described?  How could the language/descriptions be improved? |
| --- |

**eBox. 8 Proportionate Unit Reduction Example**

| John reports a monthly expenditure of €240 for his 3 chronic conditions (asthma, diabetes, and multiple/other conditions). In the hypothetical scenario, John has an unexpected tax bill, meaning that after he has spent money on essentials (rent/mortgage, food, heat etc.) there is not enough left to pay for his usual level of healthcare utilisation. He now has 25% less than his usual payments, that is €180.  Across the three chronic conditions (asthma, diabetes, and multiple/other conditions), he reduces his overall expenditure by €62.50. For asthma, he reduced his expenditure from €60 to €22.50. His percentage reduction here would be 62.5% (1-(22.5/60)). For diabetes, he reduced his expenditure from €80 to €70. His percentage reduction here would be 12.5% (1-(70/80)). For multiple/other conditions, he reduce his expenditure from €50 to €35. His percentage reduction here would be 30% (1-(35/50)). Then by adding these percentages, we see that his aggregate percentage reduction is 105%. If this reduction were distributed evenly, it would be 35% (105/3) for each condition. Therefore, the reduction for asthma for this individual would be assigned a figure of -0.79 as a 62.5% reduction represents a proportionate unit reduction for the condition of 0.79 (62.5%/35%) greater than expected. The reduction for diabetes would be assigned a figure of 0.64 as a 12.5% reduction represents a proportionate unit reduction for the condition of 0.64 (1-12.5%/35%) less than expected. The reduction for multiple/other conditions would be assigned a figure of 0.14 as a 30% reduction represents a proportionate unit reduction for the condition of 0.14 (1-30%/35%) more than expected. |
| --- |
