# Supplementary material for: How do people with multimorbidity prioritise healthcare when faced with tighter financial constraints? A national survey with a choice experiment component
Source: BMC Prim Care. 2025 Feb 27;26:57. doi: 10.1186/s12875-025-02738-9 (PMC11866811; doi:10.1186/s12875-025-02738-9)
Supplement: Supplementary file 3 — Supplementary Material 3 [file 12875_2025_2738_MOESM3_ESM.docx]

**Appendix - Screener Questionnaire**

Hello __________

We are conducting a project with researchers from the REDACTED. We are contacting you to ask if you would be interested in participating in a study researching how people with chronic medical conditions prioritise their healthcare. Participation involves an online survey that will take approximately 30 minutes. It can be completed on your smart-phone, laptop or tablet, whichever suits you best. Please read the attached information sheet for more information.

You are eligible to participate if a doctor has ever told you that you have a chronic medical condition (health problems that require ongoing management over a period of years or decades).

If you think you are eligible, we will carry out a short screening survey to ensure that you meet all criteria.

Best wishes,

***Primary Questions***

Q.1 Has a doctor ever told you that you have one of the following conditions? **SHOW CARD A. MULTICODE.**

**IF NO CHRONIC CONDITIONS, CLOSE INTERVIEW AND RE-RECRUIT.**

| **Respiratory** | |
| --- | --- |
| Chronic lung disease such as chronic bronchitis or emphysema |  |
| Asthma |  |
| **Musculoskeletal/bone disease** | |
| Arthritis (including osteoarthritis, or rheumatism) |  |
| Osteoporosis, sometimes called thin or brittle bones |  |
| **Cancer** | |
| Cancer or a malignant tumour (including leukaemia or lymphoma but excluding minor skin cancers) |  |
| **Neurological** | |
| Parkinson's disease |  |
| Alzheimer's disease |  |
| Dementia, organic brain syndrome, senility |  |
| Serious memory impairment |  |
| **Mental Health** | |
| Any emotional, nervous or psychiatric problems, such as depression or anxiety |  |
| Alcohol or substance abuse |  |
| **Gastrointestinal (Gastric Conditions)** | |
| Stomach ulcers |  |
| Cirrhosis, or serious liver damage |  |
| **Diabetes or high blood sugar (Endocrine)** | |
| Diabetes or high blood sugar |  |
| Thyroid disease |  |
| **Cardiovascular (Heart)** | |
| High blood pressure or hypertension |  |
| Angina |  |
| A heart attack (including myocardial infarction or coronary thrombosis) |  |
| Congestive heart failure (heart failure) |  |
| High cholesterol |  |
| A heart murmur |  |
| An abnormal heart rhythm |  |
| Any other heart trouble (specify)………………………… |  |
| **Vascular/Veins** | |
| Varicose Ulcers (an ulcer due to varicose veins) |  |
| A stroke (cerebral vascular disease) |  |
| Ministroke or TIA (transient ischemic attack) |  |
| Peripheral vascular disease |  |
| **Eye disease** | |
| Cataracts |  |
| Glaucoma |  |
| Blurred vision or no vision (Age related macular degeneration) |  |
| Other eye disease (specify) |  |
| None of these |  |

Q.2 Which age range do you fall into?

**IF UNDER 40 YEARS OLD, CLOSE AND RE-RECRUIT**

18-24………………………………………………… 1

25-29………………………………………………… 2

30-34………………………………………………… 3

35-39………………………………………………… 4

40-44 5

45-49 6

50-54 7

55-59 8

60-64 9

65-69 10

70-74 11

75-79 12

80-84 13

85-89 14

90+ 15

Q.3 Do you have access to a functional broadband connection?

**IF NO BROADBAND CONNECTION, CLOSE AND RE-RECRUIT.**

Yes 1

No 2

Q.4 Do you have access to a laptop/tablet/smart phone?

**IF NO ACCESS, CLOSE AND RE-RECRUIT.**

Yes 1

No 2

***Secondary Questions***

Q.5 Are you? **READ OUT. SINGLE CODE**

Male 1

Female 2

Other 3

Prefer not to say 4

Q.6 What county do you live in? **SINGLE CODE.**

| [Carlow](https://www.geni.com/projects/County-Galway-Ireland-Main-Page/17687) | 1 |
| --- | --- |
| [Cavan](https://www.geni.com/projects/County-Sligo-Ireland-Main-Page/17720) | 2 |
| [Clare](https://www.geni.com/projects/County-Wexford-Ireland-Main-Page/17772) | 3 |
| [Cork](https://www.geni.com/projects/County-Wicklow-Ireland-Main-Page/17774) | 4 |
| [Donegal](https://www.geni.com/projects/County-Louth-Ireland-Main-Page/17708) | 5 |
| [Dublin](https://www.geni.com/projects/County-Dublin-Ireland-Main-Page/17680) | 6 |
| [Galway](https://www.geni.com/projects/County-Clare-Ireland-Main-Page/17674) | 7 |
| [Kerry](https://www.geni.com/projects/County-Tipperary-Ireland-Main-Page/17765) | 8 |
| [Kildare](https://www.geni.com/projects/County-Donegal-Ireland-Main-Page/17678) | 9 |
| [Kilkenny](https://www.geni.com/projects/County-Roscommon-Ireland-Main-Page/17718) | 10 |
| [Laois](https://www.geni.com/projects/County-Laois-Queens-Irelend-Main-Page/17698) | 11 |
| [Leitrim](https://www.geni.com/projects/County-Kerry-Ireland-Main-Page/17692) | 12 |
| [Limerick](https://www.geni.com/projects/County-Kilkenny-Ireland-Main-Page/17696) | 13 |
| [Longford](https://www.geni.com/projects/County-Longford-Ireland-Main-Page/17705) | 14 |
| [Louth](https://www.geni.com/projects/County-Leitrim-Ireland-Main-Page/17700) | 15 |
| [Mayo](https://www.geni.com/projects/County-Cork-Ireland-Main-Page/17676) | 16 |
| [Meath](https://www.geni.com/projects/County-Kildare-Ireland-Main-Page/17694) | 17 |
| [Monaghan](https://www.geni.com/projects/County-Waterford-Ireland-Main-Page/17767) | 18 |
| [Offaly](https://www.geni.com/projects/County-Offaly-Kings-Ireland-Main-Page/17716) | 19 |
| [Roscommon](https://www.geni.com/projects/County-Westmeath-Ireland-Main-Page/17769) | 20 |
| [Sligo](https://www.geni.com/projects/County-Meath-Ireland-Main-Page/17712) | 21 |
| [Tipperary](https://www.geni.com/projects/County-Monaghan-Ireland-Main-Page/17714) | 22 |
| [Waterford](https://www.geni.com/projects/County-Cavan-Ireland-Main-Page/17672) | 23 |
| [Westmeath](https://www.geni.com/projects/County-Mayo-Ireland-Main-Page/17710) | 24 |
| [Wexford](https://www.geni.com/projects/County-Limerick-Ireland-Main-Page/17703) | 25 |
| [Wicklow](https://www.geni.com/projects/County-Carlow-Ireland-Main-Page/17656) | 26 |

Q.7 What is the occupation of the MAIN INCOME EARNER in your household? List below. **SINGLE CODE**

**INTERVIEWER NOW CODE HOUSEHOLD SOCIAL CLASS**

| AB | 1 |
| --- | --- |
| C1 | 2 |
| C2 | 3 |
| DE | 4 |
| F | 5 |

Q.8 Do you have a full medical card? **SINGLE CODE**.

Yes 1

No 2

Q.9 Do you have a GP visit card? **SINGLE CODE.**

Yes 1

No 2

Q.10 Do you have private insurance? **SINGLE CODE.**

Yes 1

No 2

***Contact Details***

Q.11 Can I get your full name please?

Q.12 What is your home address?

Q.13 What is your mobile phone number?

Q.14 What is your email address?

**FOR RESEARCHER’S USE:**

Interviewer Name:

Assignment Number:
