# Supplementary material for: How do people with multimorbidity prioritise healthcare when faced with tighter financial constraints? A national survey with a choice experiment component
Source: BMC Prim Care. 2025 Feb 27;26:57. doi: 10.1186/s12875-025-02738-9 (PMC11866811; doi:10.1186/s12875-025-02738-9)
Supplement: Supplementary file 4 — Supplementary Material 4 [file 12875_2025_2738_MOESM4_ESM.docx]

**eTable 1. Demographic breakdown of online and face-to-face participants**

|  | **Online (N)** | **Online (%)** | **Face to face (N)** | **Face to face (%)** | **Total (N)** |
| --- | --- | --- | --- | --- | --- |
| **Total** | **837** |  | **125** |  | **962** |
| Male | 379 | 45% | 56 | 45% | 435 |
| Female | 458 | 55% | 69 | 55% | 527 |
| **Age** |  |  |  |  |  |
| 40-49 | 219 | 26% | 0 | - | 219 |
| 50-59 | 307 | 37% | 4 | 3% | 311 |
| 60-69 | 244 | 29% | 43 | 34% | 287 |
| 70+ | 67 | 8% | 78 | 62% | 145 |
| **Social Class** |  |  |  |  |  |
| ABC1 | 436 | 52% | 30 | 24% | 466 |
| C2DE | 401 | 48% | 95 | 76% | 496 |
| **Region** |  |  |  |  |  |
| Dublin | 234 | 28% | 13 | 10% | 247 |
| Rest of Leinster | 210 | 25% | 30 | 24% | 240 |
| Munster | 230 | 27% | 59 | 47% | 289 |
| Connaught/Ulster | 163 | 19% | 23 | 18% | 186 |

**eTable 2. Prevalence of Chronic Conditions**

|  | TILDA | Choice Experiment | Physical or mental |
| --- | --- | --- | --- |
| Condition |  | % (N) |  |
| Hypertension | 37.6% (2,131) | 35.9% (N=345) | Physical |
| High Cholesterol | 36.8% (2,087) | 33.8% (N=325) | Physical |
| Arthritis | 35.7% (2,026) | 26.9% (N=259) | Physical |
| Emotional/Psychological Condition including Anxiety and Depression | 7.5% (423) | 25.7% (N=247) | Mental |
| Chronic Respiratory Disease | 11.2% (637) | 23.4% (N=225) | Physical |
| Cardiac Condition | 14.2% (805) | 14.7% (N=141) | Physical |
| Eye Disease | 17.1% (970) | 14.1% (N=136) | Physical |
| Diabetes | 9.0% (512) | 12.9% (N=124) | Physical |
| Cancer | 2.7% (152) | 8.6% (N=83) | Physical |
| Thyroid Problems | 9.2% (524) | 8.6% (N=83) | Physical |
| Gastrointestinal Conditions including Stomach Ulcers | 2.9% (163) | 8.4% (N=81) | Physical |
| Osteoporosis | 15.9% (903) | 7.3% (N=70) | Physical |
| Vascular Disease | 1.2% (69) | 3.8% (N=37) | Physical |
| Liver Disease | 0.7% (42) | 3.7% (N=36) | Physical |
| Varicose Veins including Varicose Ulcers | 1.7% (96) | 1.7% (N=16) | Physical |
| Cognitive Impairment | N.A. (<30) | 0.6% (N=6) | Physical |
| Parkinson’s Disease | 0.6% (36) | 0.3% (N=3) | Physical |

**eTable 3. Demographic and entitlement characteristics of TILDA sample with one or more condition and current sample aged 50+ years**

|  | TILDA sample with 1+ condition (N=4,919) | Current sample of those aged 50+ years  (N=743) | Chi Square |
| --- | --- | --- | --- |
|  |  |  |  |
|  | % (N) | % (N) | Chi square statistic (p value) |
| Age (years)* |  |  | 348.18 (<.001) |
| 50-59 | 15.4 (759) | 41.8 (311) |  |
| 60-69 | 39.1 (1,922) | 38.6 (287) |  |
| 70-79 | 30.4 (1,493) | 15.2 (113) |  |
| 80-89 | 13.5 (665) | 3.2 (24) |  |
| 90+ | 1.6 (80) | 1.1 (8) |  |
| Sex |  |  | 7.89 (0.02) |
| Female | 57.2 (2,816) | 55.0 (409) |  |
| Male | 42.8 (2,103) | 45.0 (334) |  |
| Location |  |  | 1.81 (0.18) |
| Urban | 55.1 (2,709) | 52.4 (389) |  |
| Rural | 44.9 (2,210) | 47.6 (354) |  |
| Private Health Insurance |  |  | 1.15 (0.28) |
| Yes | 59.4 (2,915) | 57.2 (425) |  |
| No | 40.6 (1,996) | 42.8 (318) |  |
| Number of chronic conditions+ |  |  | 1.24 (0.54) |
| 1 | 29.8 (1,466) | 31.1 (231) |  |
| 2 | 28.1 (1,381) | 28.9 (215) |  |
| 3+ | 42.1 (2,072) | 40.0 (297) |  |

* Percentages are revised for current sample to exclude those aged 40-49 years (age) because they were not samples in the TILDA study

^+^ Percentages are revised for current sample to exclude those with 0 conditions (number of chronic conditions) because they were not samples in the current study

**eTable 4. Demographic and entitlement characteristics of Choice Experiment sample aged over 50 years with one or more condition**

|  | Overall (N=743)  % (N) | One condition (N=231)  % (N) | Multimorbidity | |
| --- | --- | --- | --- | --- |
|  |  |  | Two conditions (N=215)  % (N) | Three or more conditions (N=297)  % (N) |
| Age (years) |  |  |  |  |
| 50-59 | 41.8 (311) | 48.2 (111) | 46.9 (101) | 33.4 (99) |
| 60-69 | 38.6 (287) | 38.1 (88) | 37.2 (80) | 40.0 (119) |
| 70-79 | 15.2 (113) | 12.1 (28) | 14.0 (30) | 18.5 (55) |
| 80-89 | 3.3 (24) | 1.7 (4) | 1.4 (3) | 5.8 (17) |
| 90+ | 1.1 (8) | 0.0 (0) | 0.4 (1) | 2.4 (7) |
| Sex |  |  |  |  |
| Female | 55.0 (409) | 48.5 (112) | 55.3 (119) | 59.9 (178) |
| Male | 45.0 (334) | 51.5 (119) | 44.7 (96) | 40.1 (119) |
| Location |  |  |  |  |
| Urban (5000+ people) | 52.4 (389) | 58.4 (135) | 48.8 (105) | 50.2 (149) |
| Rural (<5000 people) | 47.6 (354) | 41.6 (96) | 51.2 (110) | 49.8 (148) |
| Private Health Insurance |  |  |  |  |
| Yes | 57.2 (425) | 56.3 (130) | 56.7 (122) | 58.2 (173) |
| No | 42.8 (318) | 43.7 (101) | 43.3 (93) | 41.8 (124) |

**eTable 5. Expenditure reductions in response to financial constraints**

|  |  | Previous month’s healthcare expenditure  Mean (*SD*) | Monthly healthcare expenditure after choices made under financial constraints  Mean (*SD*) | Reduction in expenditure Mean (SD) | Mean percentage reduction in expenditure |
| --- | --- | --- | --- | --- | --- |
| Overall | GP (n=473) | €75.68 (46.69) | €46.69 (60.06) | €28.99 (48.37) | 38.3% |
|  | Medicines (n=709) | €45.69 (54.85) | €34.38 (43.38) | €11.18 (25.44) | 24.8% |
|  | Primary Care (physio, occupational therapist, psychologist) (n=151) | €86.03 (123.92) | €46.74 (74.17) | €39.30 (81.58) | 45.7% |
|  | ‘Other Healthcare (hospital visits, specialist doctors, etc.)’ (N=231) | €196.37 (634.82) | €97.75 (407.26) | €98.62 (285.75) | 50.2% |
| One Condition | GP (n=124) | €59.52 (42.28) | €40.73 (31.97) | €18.79 (27.67) | 31.6% |
|  | Medicines (n=210) | €31.52 (34.80) | €23.11 (29.64) | €8.41 (14.80) | 26.7% |
|  | Primary Care (n=37) | €49.59 (75.16) | €38.95 (60.67) | €10.65 (46.09) | 21.5% |
|  | ‘Other Healthcare’ (n=60) | €129.78 (174.66) | €60.06 (112.77) | €69.72 (121.43) | 53.7% |
| Two Conditions | GP (N=153) | €78.70 (91.33) | €47.94 (64.56) | €30.75 (50.28) | 39.1% |
|  | Medicines (n=216) | €42.80 (44.34) | €33.49 (37.26) | €9.31 (18.53) | 21.8% |
|  | Primary Care (n=44) | €99.18 (137.00) | €40.05 (60.17) | €59.14 (97.03) | 59.6% |
|  | ‘Other Healthcare’ (n=63) | €111.25 (151.69) | €50.98 (82.77) | €60.27 (103.96) | 54.2% |
| Three or  more Conditions | GP (n=196) | €85.55 (100.07) | €49.48 (69.32) | €34.07 (55.82) | 42.2% |
|  | Medicines (n=283) | €58.41 (69.56) | €43.75 (53.21) | €14.66 (34.35) | 25.1% |
|  | Primary Care (n=70) | €97.03 (133.40) | €55.06 (87.47) | €41.97 (82.43) | 43.3% |
|  | ‘Other Healthcare’ (n=108) | €283.02 (906.52) | €145.98 (584.05) | €137.04 (397.87) | 48.4% |

**eTable 6. Reaching Drug Payments Scheme Limits**

|  | % of those who spend on medication (N) |
| --- | --- |
| Medical Card & €20 limit | 2.3% (16) |
| Non-Medical Card & €100 limit | 2.8% (19) |
| Non-Medical Card & €114 limit | 1.2% (8) |
| Total | 6.2% (43) |

**eTable 7. Areas informing healthcare prioritisation decisions**

|  | Median (IQR) | 1 (Not important at all) % (N) | 2 (Not important)  % (N) | 3 (Neither important nor not important  % (N) | 4 (Important)  % (N) | 5 (Very important)  % (N) |
| --- | --- | --- | --- | --- | --- | --- |
| Maintaining Independence | 5 (4-5) | 0.9  (9) | 2.7  (26) | 8.7  (84) | 22.9 (220) | 64.8  (623) |
| Symptom Control | 5 (4-5) | 0.2  (2) | 0.9  (9) | 6.0 (58) | 21.3 (205) | 71.5  (688) |
| Doctors’ Advice | 4 (4-5) | 1.8  (17) | 4.7 (45) | 15.8 (152) | 28.6 (275) | 49.2  (473) |
| Staying Alive | 5 (5-5) | 0.5  (5) | 0.8  (8) | 5.0 (48) | 10.8 (104) | 82.8  (797) |
| Treatment Burden | 4 (3-5) | 5.2  (50) | 9.4 (90) | 20.5 (197) | 29.2 (281) | 35.8  (344) |

**eTable 8. Content analysis of ‘other’ factors considered when deciding which healthcare areas to reduce expenditure**

|  | % (N) |
| --- | --- |
| Intensity of illness(es) at the given time | 2.6% (25) |
| Family advice | 0.7% (7) |
| Other responsibilities | 2.2% (20) |
| Availability of alternative care/therapy | 7.2% (69) |
| COVID-19 | 0.4% (4) |
| Ability to pay at a later date | 0.4% (4) |
| Perceived value for money of care/therapy | 1.1% (11) |
| Uncategorised | 1.3% (13) |

**eTable 9. Distribution of hypothetical likelihood of invoking cost saving measures**

|  | 1 (Very Unlikely)  % (N) | 2 (Unlikely)  % (N) | 3 (Neither unlikely nor likely)  % (N) | 4 (Likely)  % (N) | 5 (Very Likely)  % (N) |
| --- | --- | --- | --- | --- | --- |
| Use Savings | 7.0%  (67) | 6.7% (64) | 12.3% (118) | 22.5% (216) | 51.7% (497) |
| Borrow Money | 35.7%  (343) | 20.1% (193) | 15.4% (148) | 13.6% (131) | 15.3% (147) |
| Not pay other bills | 31.4%  (302) | 18.0% (173) | 20.4% (196) | 17.4% (167) | 12.9% (124) |
| Cut down food spending | 12.9%  (124) | 12.4% (119) | 23.8% (229) | 27.0% (260) | 23.9% (230) |
| Cut down recreational spending | 5.2%  (50) | 4.1% (39) | 8.8% (85) | 15.6% (150) | 66.3% (638) |
| Cut down general expenses | 3.7%  (36) | 4.9% (47) | 12.2% (117) | 25.1% (241) | 54.2% (521) |
| Sacrifice usual healthcare usage | 18.6%  (179) | 13.2% (127) | 22.1% (213) | 25.5% (245) | 20.6% (198) |

**eTable 10. Content analysis of ‘other’ alternatives to reducing healthcare expenditure**

|  | % (N) |
| --- | --- |
| Increase income (e.g. sell assets or work more) | 4.7% (45) |
| Negotiate with healthcare provider on price | 0.7% (7) |
| Substitute for other form of care (e.g. teleconsultation) | 1.9% (18) |
| Set-up a payment plan | 0.2% (2) |
| Access care in other country | 0.4% (4) |
| Seek out healthcare entitlements | 1.4% (13) |
| Seek help from charity | 0.3% (3) |
| Improve health (e.g. through exercise, diet etc.) | 3.4% (33) |
| Shop around for better price | 0.7% (7) |
| Stop/start/maintain private health insurance | 2.0% (19) |
| Uncategorised | 0.4% (4) |

**eTable 11. Distribution of worry about financial problems caused by healthcare**

|  | 1 (not at all)  % (N) | 2  % (N) | 3  % (N) | 4  % (N) | 5  % (N) | 6  % (N) | 7 (Very Much)  % (N) |
| --- | --- | --- | --- | --- | --- | --- | --- |
| Worry about financial problems caused by healthcare | 19.6%  (189) | 14.3% (138) | 12.1% (116) | 13.0% (125) | 13.4%  (129) | 12.6% (121) | 15.0%  (144) |
