# Supplementary material for: How do people with multimorbidity prioritise healthcare when faced with tighter financial constraints? A national survey with a choice experiment component
Source: BMC Prim Care. 2025 Feb 27;26:57. doi: 10.1186/s12875-025-02738-9 (PMC11866811; doi:10.1186/s12875-025-02738-9)
Supplement: Supplementary file 5 — Supplementary Material 5 [file 12875_2025_2738_MOESM5_ESM.docx]

**Appendix J – Outlier Management**

**Cost related non-attendance**

**Question**

Q9 In the last twelve months, have you ever not attended a healthcare professional when you thought you needed to, because of the cost of the visit (or the costs associated with the visit such as travel, taking time off work etc.)?

Yes/no Number of Occasions _____

**Results**

For healthcare visits, 31.1% (N=299) of participants reported not attending a healthcare professional in the previous 12 months because of costs, and among those that did not attend, the average number of occasions that this occurred in the previous 12 months was 2.4 (SD=1.8).

**Possible Outliers:**

| Row number | Q9 HCP Number of Occasions | Q11 Medicines Number of Occasions (for context) |  |
| --- | --- | --- | --- |
| 304 | 50 | 50 | Not feasible – – remove entry |
| 625 | 20 | 30 | Not feasible – remove entry |
| 695 | 12 | 5 | Feasible |
| 835 | 12 | 24 | Feasible |

**Cost related non-adherence**

**Question**

Q11 In the last twelve months, have you ever not purchased medication you needed for your treatment because of the cost?

Yes/no Number of Occasions _____

**Results**

For cost-related non-adherence to medications, 15.5% (N=149) of participants reported not buying a medication in the previous 12 months because they could not afford it, and among those that did not buy a medication, the average number of occasions that this occurred in the previous 12 months was 2.7 (SD=2.9).

**Possible Outliers:**

| Row number | Q9 HCP Number of Occasions (for context) | Q11 Medicines Number of Occasions | Suggested action |
| --- | --- | --- | --- |
| 304 | 50 | 50 | Not feasible – remove entry |
| 625 | 20 | 30 | Not feasible – remove entry |
| 694 | 0 | 50 | Not feasible – remove entry |
| 835 | 12 | 24 | Feasible |

**Expenditure**

**Question**

**Question 3.1.1**

In the boxes below, please estimate your use of healthcare for the last month and also how much each aspect of this healthcare use cost you (including travel expenses such as parking and fuel for journeys to pharmacy, GP or hospital)

COST OF €0 ALLOWED

| **Healthcare Service** | **Number** | **Total Cost in Last Month** |
| --- | --- | --- |
| ***GP Visits*** |  |  |
| GP Visit – **CODE FROM Q1.1.1** (e.g Diabetes) |  |  |
| GP Visit – **CODE FROM Q1.1.1** (e.g Depression) |  |  |
| GP Visit – Multiple/other illnesses |  |  |
| ***Medicines*** |  |  |
| Medicines – **CODE FROM Q1.1.1** (e.g Diabetes) |  |  |
| Medicines – **CODE FROM Q1.1.1** (e.g Depression) |  |  |
| Medicines – Multiple/other illnesses |  |  |
| ***Primary Care (physio, occupational therapist, psychologist) Visits*** |  |  |
| Primary Care Visits – **CODE FROM Q1.1.1** (e.g Diabetes) |  |  |
| Primary Care Visits – **CODE FROM Q1.1.1** (e.g Depression) |  |  |
| Primary Care Visits – Multiple/other illnesses |  |  |
| ***Other Healthcare (hospital visits, specialist doctors, etc) Visits*** |  |  |
| Other Healthcare Visits – **CODE FROM Q1.1.1** (e.g Diabetes) |  |  |
| Other Healthcare Visits – **CODE FROM Q1.1.1** (e.g Depression) |  |  |
| Other Healthcare Visits – Multiple/other illnesses |  |  |
| **Total Cost in Last Month** |  |  |

**Results**

|  |  | Previous month’s healthcare expenditure  Mean (*sd*) |
| --- | --- | --- |
| Overall | GP | €47.88 (163.61) |
|  | Medicines | €44.05 (161.37) |
|  | Primary Care | €14.07 (59.90) |
|  | Other Healthcare | €76.07 (926.53) |

**Possible Outliers:**

| Row number | GP Visits | No. Medicines | Primary Care Visits | Other Visits | GP Cost | Medicine Cost | Primary Care Cost | Other Cost | Suggested action |
| --- | --- | --- | --- | --- | --- | --- | --- | --- | --- |
| 156 | 3 | 4 | 0 | 0 | 555 | 125 | 0 | 0 | Gp exp error – remove |
| 205 | 1 | 25 | 0 | 2 | 500 | 2010 | 0 | 27000 | All errors - remove |
| 241 | 4 | 1 | 0 | 0 | 545 | 75 | 0 | 0 | Feasible amount |
| 263 | 10 | 14 | 0 | 0 | 550 | 295 | 0 | 0 | GP Not feasible - remove |
| 346 | 6 | 3 | 7 | 6 | 432 | 32 | 815 | 947 | Feasible amount |
| 396 | 0 | 12 | 0 | 0 | 0 | 1200 | 0 | 0 | Med exp Not feasible - remove |
| 435 | 2 | 14 | 0 | 3 | 110 | 66 | 0 | 2000 | Feasible |
| 448 | 6 | 10 | 10 | 7 | 177 | 3803 | 307 | 225 | Medicine exp error – remove  Primary care - feasible |
| 461 | 14 | 14 | 0 | 2 | 3800 | 600 | 0 | 0 | Gp exp error – remove GP entry  Medicines exp – remove meds entry |
| 497 | 23 | 11 | 8 | 4 | 700 | 270 | 200 | 115 | Gp exp error - remove |
| 579 | 4 | 4 | 0 | 0 | 0 | 600 | 0 | 0 | Medicine exp error – remove |
| 694 | 47 | 5 | 0 | 0 | 500 | 0 | 0 | 0 | Gp exp error - remove |
| 823 | 7 | 5 | 5 | 5 | 550 | 536 | 870 | 830 | feasible |
| 857 | 0 | 1 | 0 | 2 | 0 | 20 | 0 | 800 | Feasible |
| 869 | 0 | 4 | 3 | 2 | 0 | 46 | 120 | 2060 | Feasible |
| 900 | 1 | 4 | 0 | 1 | 60 | 228 | 0 | 9000 | Feasible |
| 914 | 5 | 4 | 0 | 0 | 2080 | 1620 | 0 | 0 | Error – remove all expenditure data |
| 921 | 51 | 60 | 23 | 17 | 630 | 140 | 459 | 898 | Remove all |
| 927 | 17 | 0 | 0 | 0 | 1000 | 0 | 0 | 0 | Remove GP |
